# Developing and validating models to predict sudden death and pump failure death in patients with heart failure and preserved ejection fraction

**DOI:** 10.1007/s00392-020-01786-8

**Published:** 2020-12-10

**Authors:** Li Shen, Pardeep S. Jhund, Inder S. Anand, Peter E. Carson, Akshay S. Desai, Christopher B. Granger, Lars Køber, Michel Komajda, Robert S. McKelvie, Marc A. Pfeffer, Scott D. Solomon, Karl Swedberg, Michael R. Zile, John J. V. McMurray

**Affiliations:** 1 Department of Medicine, Hangzhou Normal University, Hangzhou, 310003 China; 2 British Heart Foundation Cardiovascular Research Centre, University of Glasgow, 126 University Place, Glasgow, G12 8TA UK; 3 Department of Medicine, University of Minnesota Medical School and VA Medical Center, Minneapolis, USA; 4 Department of Cardiology, Washington VA Medical Center, Washington, DC USA; 5 Department of Medicine, Brigham and Women’s Hospital, Harvard Medical School, Boston, USA; 6 Duke Clinical Research Institute, Duke University, Durham, NC USA; 7 Rigshospitalet Copenhagen University Hospital, Copenhagen, Denmark; 8 Department of Cardiology, Hospital Saint Joseph, Paris, France; 9 Western University, London, ON Canada; 10 Department of Molecular and Clinical Medicine, University of Gothenburg, Gothenburg, Sweden; 11 Medical University of South Carolina and RHJ Department of Veterans Administration Medical Center, Charleston, USA

**Keywords:** Heart failure, Sudden death, Pump failure death, Risk, Model

## Abstract

**Background:**

Sudden death (SD) and pump failure death (PFD) are leading modes of death in heart failure and preserved ejection fraction (HFpEF). Risk stratification for mode-specific death may aid in patient enrichment for new device trials in HFpEF.

**Methods:**

Models were derived in 4116 patients in the Irbesartan in Heart Failure with Preserved Ejection Fraction trial (I-Preserve), using competing risks regression analysis. A series of models were built in a stepwise manner, and were validated in the Candesartan in Heart failure: Assessment of Reduction in Mortality and morbidity (CHARM)-Preserved and Treatment of Preserved Cardiac Function Heart Failure with an Aldosterone Antagonist (TOPCAT) trials.

**Results:**

The clinical model for SD included older age, men, lower LVEF, higher heart rate, history of diabetes or myocardial infarction, and HF hospitalization within previous 6 months, all of which were associated with a higher SD risk. The clinical model predicting PFD included older age, men, lower LVEF or diastolic blood pressure, higher heart rate, and history of diabetes or atrial fibrillation, all for a higher PFD risk, and dyslipidaemia for a lower risk of PFD. In each model, the observed and predicted incidences were similar in each risk subgroup, suggesting good calibration. Model discrimination was good for SD and excellent for PFD with Harrell’s C of 0.71 (95% CI 0.68–0.75) and 0.78 (95% CI 0.75–0.82), respectively. Both models were robust in external validation. Adding ECG and biochemical parameters, model performance improved little in the derivation cohort but decreased in validation. Including NT-proBNP substantially increased discrimination of the SD model, and simplified the PFD model with marginal increase in discrimination.

**Conclusions:**

The clinical models can predict risks for SD and PFD separately with good discrimination and calibration in HFpEF and are robust in external validation. Adding NT-proBNP further improved model performance. These models may help to identify high-risk individuals for device intervention in future trials.

**Clinical trial registration:**

I-Preserve: ClinicalTrials.gov NCT00095238; TOPCAT: ClinicalTrials.gov NCT00094302; CHARM-Preserved: ClinicalTrials.gov NCT00634712.

**Graphic abstract:**

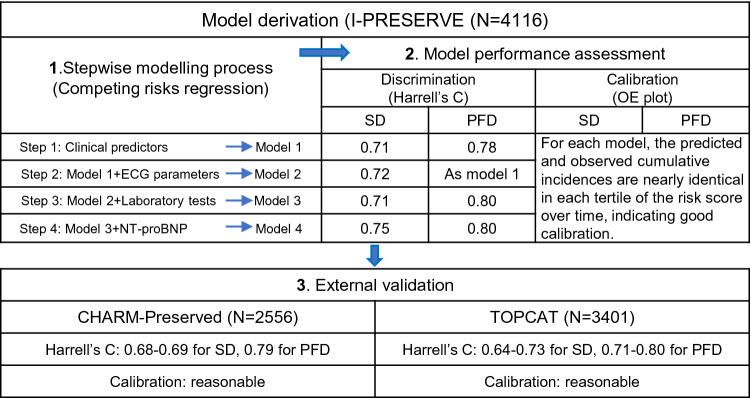

**Supplementary Information:**

The online version contains supplementary material available at 10.1007/s00392-020-01786-8.

## Introduction

In heart failure, implantable cardioverter defibrillators (ICDs) are currently indicated as a primary preventive therapy only in selected patients with reduced ejection fraction (HFrEF) [1, 2]. Yet, sudden death is also relatively common in patients with heart failure and preserved ejection fraction (HFpEF), and the potential value of an ICD in patients with HFpEF has been discussed [3–5]. However, the cause of death in individual patients with HFpEF is highly variable, and if any future trial of ICD therapy is to be successful (and device therapy in HFpEF to be affordable), identification of the subgroup of patients at highest risk of sudden death is essential. Unfortunately, there is a paucity of studies on the prediction of mode of death in patients with HFpEF, with only one predictive model for sudden death [6]. Despite good discriminative ability, this existing model did not take account of the competing risk of death from other causes, which is considerable in patients with HFpEF, and it has not been validated in an independent cohort [6]. Therefore, the aim of this study was to develop models to predict sudden death and pump failure death, separately, using standard demographic and clinical variables, along with ECG findings and laboratory measurements including N-terminal pro-B-type natriuretic peptide (NT-proBNP). These models were developed in the Irbesartan in Heart Failure with Preserved Ejection Fraction Study (I-Preserve) [7] and validated in similar patients enrolled in the Candesartan in Heart failure: Assessment of Reduction in Mortality and morbidity (CHARM)-Preserved study and the Treatment of Preserved Cardiac Function Heart Failure with an Aldosterone Antagonist (TOPCAT) study [8, 9].

## Methods

### The derivation and validation cohorts

This study consisted of one derivation cohort of patients with HFpEF (I-Preserve) and two validation cohorts (CHARM-Preserved and TOPCAT) [7–9]. The design and primary results of these trials have been previously reported. Briefly, I-Preserve randomized 4128 patients aged ≥ 60 years who had symptomatic (New York Heart Association [NYHA] class II–IV) HF with a left-ventricular ejection fraction (LVEF) ≥ 45% to receive irbesartan or placebo. Patients were required to have current symptoms and signs of HF and corroborating evidence including pulmonary congestion on radiography, left-ventricular hypertrophy (LVH) or left atrial enlargement on echocardiography, or LVH or left bundle branch block (LBBB) on ECG. Patients in NYHA class II were additionally required to have had an HF hospitalization within the past 6 months [7]. CHARM-Preserved compared candesartan with placebo in 3023 patients who had NYHA class II–IV HF with an LVEF > 40%, and patients in NYHA class II were required to have been hospitalized for a cardiac reason within the past 6 months [9]. TOPCAT enrolled 3445 patients aged ≥ 50 years who had symptomatic HF with an LVEF ≥ 45%; patients were eligible if they had been hospitalized for HF within the past 12 months, or had an elevated natriuretic peptide level within 60 days before randomization (i.e., B-type natriuretic peptide [BNP] ≥ 100 pg/ml or NT-proBNP ≥ 360 pg/ml) [8]. Patients having an ICD or cardiac resynchronization therapy with defibrillator (CRT-D) at baseline (if any) were excluded, as these devices selectively reduce the risk of one of the two modes of death of interest. Patients with an LVEF < 45% in CHARM-Preserved were excluded to ensure a consistent LVEF entry threshold across the trials. Each trial was approved by the ethics committee at participating centers and all patients provided written informed consent.

### Outcomes of interest

Sudden death and pump failure death were the outcomes of interest in the study. In each trial, all deaths were adjudicated by a clinical endpoint committee in a blinded fashion according to pre-specified criteria. Similar definitions for mode-specific death were used across these trials: sudden death was defined as an unexpected death in an otherwise stable patient, and pump failure death was defined as a death occurring within the context of clinically worsening symptoms and/or signs of HF without evidence of another cause of death (definitions in detail are presented in Online Table 1).

### Candidate prediction variables

A wide range of baseline variables (*N* = 45) were assessed to identify predictors for sudden death and pump failure death separately in I-Preserve. These variables included demographics, clinical variables, medical history, ECG parameters, routine laboratory tests, and NT-proBNP. A full set of baseline variables was collected in most patients in I-Preserve (with missing observations < 5%), except for NT-proBNP which was measured in 84% of the cohort. The clinical variables were available in most patients in both validation cohorts, but the laboratory measurements were not made in CHARM-Preserved, except for serum creatinine/eGFR which was available in the patients from North America (39% of the cohort). In TOPCAT, the required laboratory measurements were recorded in most patients (missing observations < 5%), except for blood urea nitrogen (available in 77% of the cohort) and NT-proBNP (available in 18%).

### Statistical analysis

For each mode of death, the prognostic value of each candidate variable on the cumulative incidence was first examined with the use of a univariate Fine-Gray regression model [10]. For each continuous variable, its linear association with the cumulative incidence of mode-specific death was examined graphically by means of the restricted cubic spline method, and if the response appeared non-linear, certain cut-off values or transformations were used based on the spline curves and clinical relevance [11]. For each categorical variable, appropriate dummy variables were applied based on literature and data availability. The statistical power for each candidate variable was quantified by Chi-square values with one degree of freedom which was positively associated with the prediction strength. For each outcome, univariate predictors significant at a *p* value < 0.20 were included in multivariable Fine-Gray regression analyses with a backward stepwise selection at an exclusion p value of 0.05. A total of four multivariable models were developed for each mode of death: Model 1 was selected from candidate variables including demographics, clinical features, and medical history; Model 2 was developed using the candidate variables for Model 1 with the addition of ECG parameters; Model 3 used routine laboratory test in addition to the variables for Model 2, and Model 4 added NT-proBNP to the variables used in Model 3. Each model was developed using the complete-case analysis method. The proportional subdistribution hazard assumption was examined using time-varying terms for the derived models.

For each derived model, an individual’s risk score was calculated as the sum of each predictor value multiplied by its corresponding coefficient from the multivariable model. Model calibration was graphically examined by comparing the predicted cumulative incidence with the observed Aalen–Johansen estimator in each tertile of the risk score (the closer the better) [12]. Model discrimination was examined by visually assessing the separation of each set of curves (the wider the better) and by computing the Harrell’s C statistic [11].

External validation was performed in CHARM-Preserved as well as in TOPCAT. In CHARM-Preserved, only Model 1 and Model 2 for mode-specific death were validated, given that the laboratory variables were not available in most patients. In TOPCAT, the four derived models for each mode of death were validated. For each model to be validated, an individual’s risk score was calculated as the sum of predictor coefficients from each derivation model multiplied by its corresponding predictor values in the validation cohort. The obtained risk score for each model was then fitted into a univariate Fine-Gray regression analysis. Model performance was examined using the same approach as the derivation procedure. Given the small cohort size of patients with NT-proBNP measurements in TOPCAT, the risk score of Model 4 for each mode of death was categorised into two subgroups by which the observed and predicted cumulative incidences over time were plotted and compared.

To examine the ability of our models to separate patients with different outcomes by the end of follow-up, the risk scores for sudden death and pump failure death at baseline were calculated, separately, for each patient in I-Preserve, using the respective models (in each case Model 4, including NT-proBNP). Thereafter, the distribution of outcomes (i.e., sudden death, pump failure death, and other deaths or alive) was plotted against both of the risk scores simultaneously.

We also compared mode-specific rates of death in high-risk patients in I-Preserve with the same rates in patients in the control groups in the Sudden Cardiac Death in Heart Failure Trial (SCD-HeFT) [13]. The patients in I-Preserve were those in the highest tertile of the sudden death risk score (based on model 4), along with the addition of age (≤ 70 vs. > 70 years) as another stratifier.

A two-tailed *p* < 0.05 was considered statistically significant. All analyses were performed using STATA software (version 14.0 SE).

## Results

### Patient characteristics and mortality events in the derivation cohort

The derivation cohort included 4116 patients with HFpEF in I-Preserve, after excluding 12 patients with an ICD or CRT-D at baseline. The average age was 72 years and 60% were women. The mean LVEF was 59%, the vast majority (97%) were in NYHA class II-III (predominantly in class III) symptoms, and most had a hypertensive etiology (64%). The baseline characteristics are shown in Table 1.

**Table 1:** Baseline characteristics of patients with HFpEF in the derivation and validation cohorts

	Derivation cohort	Validation cohorts	
	I-Preserve	CHARM-Preserved	TOPCAT
	(*N* = 4116)	(*N* = 2556)	(*N* = 3401)
Age-years	71.6 ± 6.9	66.9 ± 11.0	68.5 ± 9.6
Female (%)	2485 (60.4)	1077 (42.1)	1760 (51.8)
Race (%)			
White	3847 (93.5)	2337 (91.4)	3028 (89.0)
Black	82 (2.0)	107 (4.2)	294 (8.6)
Asian	35 (0.9)	64 (2.5)	18 (0.5)
Other	152 (3.7)	48 (1.9)	61 (1.8)
Blood pressure—mmHg			
Systolic	136.4 ± 15.0	136.6 ± 18.6	129.3 ± 13.9
Diastolic	78.8 ± 9.1	77.8 ± 10.7	75.9 ± 10.6
Heart rate—beats/min	71.4 ± 10.5	71.4 ± 12.4	69.0 ± 10.6
Body mass index	29.6 ± 5.3	29.3 ± 5.8	32.1 ± 7.1
LVEF—%	59.4 ± 9.2	56.1 ± 8.7	57.1 ± 7.4
NYHA class (%)			
I–II	869 (21.1)	1582 (61.9)	2282 (67.2)
III–IV	3246 (78.9)	974 (38.1)	1116 (32.8)
Etiology (%)			
Ischemic	1033 (25.1)	1378 (53.9)	–
Hypertensive	2616 (63.6)	631 (24.7)	–
Other	467 (11.3)	547 (21.4)	–
Medical history (%)			
Current smoking	–	328 (12.8)	357 (10.5)
HF hospitalization within last 6 months	1809 (44.0)	935 (36.6)	1787 (52.5)
Myocardial infarction	963 (23.4)	1046 (40.9)	873 (25.7)
Angina	1773 (43.1)	1509 (59.0)	1598 (47.0)
CABG or PCI	542 (13.2)	821 (32.1)	791 (23.3)
Coronary artery disease	2087 (50.7)	1790 (70.0)	1993 (58.6)
Hypertension	3645 (88.6)	1683 (65.8)	3109 (91.5)
Diabetes	1128 (27.4)	727 (28.4)	1096 (32.3)
Atrial fibrillation	1199 (29.1)	762 (29.8)	1192 (35.1)
Stroke	394 (9.6)	222 (8.7)	260 (7.7)
Pacemaker	245 (6.0)	183 (7.2)	247 (7.3)
COPD or asthma	386 (9.4)	–	543 (16.0)
Dyslipidemia	1801 (43.8)	–	2039 (60.0)
Treatment (%)			
Digitalis	556 (13.5)	680 (26.6)	337 (9.9)
Diuretics	3407 (82.8)	1909 (74.7)	2778 (81.9)
Loop	2140 (52.0)	1576 (61.7)	1764 (52.0)
Thiazide	1552 (37.7)	355 (13.9)	1394 (41.1)
ACEI or ARB	2572 (62.5)	1499 (58.6)	2863 (84.2)
Beta-blocker	2423 (58.9)	1405 (55.0)	2637 (77.7)
MRA	631 (15.3)	302 (11.8)	1698 (49.9)
Calcium channel blocker	1634 (39.7)	833 (32.6)	1284 (37.8)
Antiarrhythmic agent	355 (8.6)	250 (9.8)	289 (8.5)
Antiplatelet	2412 (58.6)	1562 (61.1)	2292 (67.6)
Oral anticoagulant	783 (19.0)	625 (24.5)	774 (22.8)
Lipid lowering agent	1272 (30.9)	1052 (41.2)	1816 (53.5)
Anti-diabetic agent	922 (22.4)	–	943 (27.8)
ECG			
QRS duration—milliseconds	90 (80–106)		92 (82–106)
Atrial fibrillation or flutter (%)	694 (16.9)	421 (16.5)	689 (20.4)
Bundle branch block (%)	613 (14.9)	346 (13.6)	589 (17.4)
Left bundle branch block (%)	336 (8.2)	–	–
Right bundle branch block (%)	283 (6.9)	-	–
Left ventricular hypertrophy (%)	1257 (30.5)	373 (14.7)	738 (21.8)
Laboratory tests			
Albumin—g/l	43.1 ± 3.4	–	41.1 ± 5.4
Aspartate aminotransferase—U/l	23.7 ± 10.5	–	25.4 ± 12.6
Alanine aminotransferase—U/l	23.3 ± 15.2	–	25.1 ± 14.3
Bilirubin—mg/dl	0.65 ± 0.29	–	0.73 ± 0.66
Potassium—mmol/l	4.44 ± 0.47	–	4.25 ± 0.45
Sodium—mmol/l	139.5 ± 3.0	–	141.2 ± 4.2
Hemoglobin—g/l	140.0 ± 15.0	–	133.0 ± 16.8
Hematocrit—%	42.1 ± 4.5	–	41.2 ± 66.3
Leukocyte—10^9^/l	7.15 ± 2.0	–	7.07 ± 3.8
Neutrophil—10^9^/l	4.53 ± 1.7	–	–
Platelet—10^9^/l	233.8 ± 66.8	–	231.6 ± 66.6
Blood urea nitrogen—mg/dl	21.3 ± 9.3	–	21.2 ± 11.3
Creatinine—mg/dl	1.00 ± 0.32^a^	1.12 ± 0.41^c^	1.09 ± 0.30
eGFR—ml/min/1.73m^2^	72.6 ± 22.5^a^	72.2 ± 27.1^c^	67.7 ± 20.2
eGFR < 60 ml/min/1.73m^2^	1239 (30.8)^a^	322 (34.9)^c^	1307 (38.5)
NT-proBNP—pg/ml	339 (133–960)^b^	–	843 (463–1727)^d^

Plus-minus values are mean ± standard deviation. QRS duration and NT-proBNP are presented as median with interquartile range. Other values are presented in number with percentage

*LVEF* left-ventricular ejection fraction, *NYHA* New York Heart Association, *CABG* coronary artery bypass grafting, *PCI* percutaneous coronary intervention, *COPD* chronic obstructive pulmonary disease, *ACEI* angiotensin-converting enzyme inhibitor, *ARB* angiotensin receptor blocker, *MRA* mineralocorticoid receptor antagonist, *eGFR* estimated glomerular filtration rate, *NT-proBNP* N-terminal pro-B-type natriuretic peptide

The letters denote the number of patients available: ^a^4027 (98%); ^b^3470 (84%); ^c^922 (39%); ^d^615 (18%)

‘–’ denotes data not available

During a median 52.9 months of follow-up, 877 death events occurred, including 230 sudden deaths and 123 pump failure deaths. The corresponding annual rates for sudden death and pump failure death were 1.4 (95% CI 1.2–1.6) and 0.7 (95% CI 0.6–0.9) per 100 patient-years, respectively.

### Derivation of the sudden death models

The 25 strongest prediction variables for sudden death were listed in a descending order of prediction strength from the univariate analysis in I-Preserve in Online Table 2; the five most powerful prognostic variables were NT-proBNP, LVEF, blood urea nitrogen, male sex, and serum creatinine.

The four multivariable models for sudden death derived from four sets of candidate variables are summarized in Table 2. Model 1 included 7 prediction variables: older age, male sex, lower LVEF, higher heart rate, history of diabetes or myocardial infarction, and HF hospitalization within previous 6 months, all of which were independently associated with a higher risk of sudden death. Model 2 further included LVH and bundle branch block on the ECG, and both were associated with a higher risk of sudden death, in addition to the predictive variables in Model 1. In Model 3, serum albumin entered the model and a lower albumin was associated with a higher risk of sudden death, but heart rate and bundle branch block on ECG dropped out of the model. In Model 4, albumin and HF hospitalization within previous 6 months dropped from the model once NT-proBNP was included, with higher levels of NT-proBNP associated with a higher risk of sudden death.

**Table 2:** Multivariable models for sudden death in I-Preserve

	Sudden death Model 1				Sudden death Model 2			
Number of patients (number of events)	4109 (230)				4109 (230)			
C statistic	0.71 (95% CI 0.68–0.75)				0.72 (95% CI: 0.69–0.75)			
	Coefficient	sHR (95% CI)	*χ*^2^	*p* value	Coefficient	sHR (95% CI)	*χ*^2^	*p* value
Age 60 years or above, per 1 year increase	0.049	1.05 (1.03–1.07)	25.5	< 0.001	0.05	1.05 (1.03–1.07)	24.8	< 0.001
Male sex	0.553	1.74 (1.33–2.27)	16.6	< 0.001	0.551	1.74 (1.33–2.27)	16.3	< 0.001
LVEF 45–60%, per 1% decrease	0.053	1.05 (1.03–1.08)	15.7	< 0.001	0.051	1.05 (1.02–1.08)	14.3	< 0.001
Heart rate 50–100 beats/min, per 5 beats/min	0.070	1.07 (1.01–1.14)	5.2	0.022	0.067	1.07 (1.01–1.13)	4.8	0.029
History of diabetes	0.519	1.68 (1.28–2.20)	14.3	< 0.001	0.531	1.70 (1.30–2.23)	14.7	< 0.001
History of myocardial infarction	0.419	1.52 (1.14–2.02)	8.3	0.004	0.435	1.54 (1.16–2.05)	9.0	0.003
HF hospitalization within previous 6 months	0.364	1.44 (1.10–1.88)	7.1	0.007	0.373	1.45 (1.11–1.89)	7.5	0.006
Bundle branch block on ECG					0.327	1.39 (1.00–1.92)	3.9	0.049
Left ventricular hypertrophy on ECG					0.376	1.46 (1.11–1.92)	7.3	0.007

	Sudden death Model 3				Sudden death Model 4			
Number of patients (number of events)	4021 (228)				3467 (195)			
C statistic	0.71 (95% CI: 0.68–0.75)				0.75 (95% CI: 0.72–0.78)			
	Coefficient	sHR (95% CI)	*χ*^2^	*p* value	Coefficient	sHR (95% CI)	*χ*^2^	*p* value
Age 60 years or above, per 1 year increase	0.046	1.05 (1.03–1.07)	21.0	< 0.001	0.034	1.03 (1.01–1.06)	9.5	0.002
Male sex	0.529	1.70 (1.30–2.22)	14.8	< 0.001	0.506	1.66 (1.23–2.23)	11.3	0.001
LVEF 45–60%, per 1% decrease	0.055	1.06 (1.03–1.08)	16.7	< 0.001	0.036	1.04 (1.01–1.07)	5.8	0.017
Heart rate 50–100 beats/min, per 5 beats/min								
History of diabetes	0.547	1.73 (1.32–2.27)	15.5	< 0.001	0.568	1.76 (1.31–2.37)	14.2	< 0.001
History of myocardial infarction	0.415	1.51 (1.14–2.01)	8.2	0.004	0.463	1.59 (1.17–2.15)	9.0	0.003
HF hospitalization within the last 6 months	0.336	1.40 (1.07–1.83)	6.0	0.014				
Bundle branch block on ECG								
Left ventricular hypertrophy on ECG	0.395	1.48 (1.13–1.95)	8.0	0.005	0.34	1.40 (1.04–1.90)	4.9	0.027
Albumin 35–45 g/l, per 1 g/l decrease	0.065	1.07 (1.01–1.12)	6.2	0.013				
NT-proBNP up to 3000 pg/ml, per 100 pg/ml					0.048	1.05 (1.03–1.06)	42.6	< 0.001

*sHR* subdistribution hazard ratio, *CI* confidence interval, *LVEF* left-ventricular ejection fraction, *ECG* electrocardiography, *NT-proBNP* N-terminal pro-B-type natriuretic peptide

*χ*^2^ score: the larger *χ*^2^ value, the more powerful the predictor

For the continuous variables, there was no further trend in the risk of sudden death with age for values < 60 years, for LVEF with values of > 60%, for albumin with values of < 35 or > 45 g/L, and for NT-proBNP with values of > 3000 pg/ml (Online Fig. A1).

### Derivation of the pump failure death models

The 25 most powerful prediction variables for pump failure death are shown in Online Table 3, in a descending order of prediction strength based on the univariate analysis, and the five strongest prediction variables were NT-proBNP, blood urea nitrogen, serum creatinine, age, and eGFR.

The four multivariable models for pump failure death are presented in Table 3. Model 1 included 8 variables, in which older age, male sex, lower LVEF or diastolic blood pressure, higher heart rate, and history of diabetes or atrial fibrillation were associated with higher risks for pump failure death, while a history of dyslipidaemia was associated with a lower risk. None of the ECG-derived candidate variables were further selected, i.e., Model 2 was identical to Model 1. Compared with Model 1, Model 3 additionally selected serum albumin, potassium, and creatinine, and a lower level of albumin and higher levels of potassium and creatinine were associated with higher risks of pump failure death. However, with the addition of these variables, sex and heart rate fell out of the model. Model 4 included NT-proBNP, and a higher level of NT-proBNP was associated with higher risk for pump failure death while LVEF, potassium, albumin, and history of atrial fibrillation fell out of the model.

**Table 3:** Multivariable models for pump failure death in I-Preserve

	Pump failure death Model 1				Pump failure death Model 2	
Number of patients (number of events)	4109 (123)					
C statistic	0.78 (95% CI: 0.75–0.82)					
	Coefficient	sHR (95% CI)	*χ*^2^	*p* value		
Age 60 years or above, per 1 year increase	0.067	1.07 (1.04–1.10)	24.9	< 0.001	Same as pump failure death Model 1	
Male sex	0.395	1.48 (1.03–2.13)	4.6	0.032		
Heart rate 50–100 beats/min, per 5 beats/min increase	0.099	1.10 (1.02–1.20)	5.5	0.019		
LVEF 45–60%, per 1% decrease	0.052	1.05 (1.02–1.09)	8.6	0.003		
Diastolic BP up to 80 mmHg, per 1 mmHg decrease	0.041	1.04 (1.02–1.07)	11.3	0.001		
History of diabetes	0.839	2.31 (1.63–3.29)	21.7	< 0.001		
History of dyslipidemia	− 0.646	0.52 (0.36–0.77)	10.6	0.001		
History of atrial fibrillation	0.593	1.81 (1.25–2.62)	9.8	0.002		

	Pump failure death Model 3				Pump failure death Model 4			
Number of patients (number of events)	3993 (116)				3448 (98)			
C statistic	0.80 (95% CI: 0.76–0.83)				0.80 (95% CI: 0.76–0.84)			
	Coefficient	sHR (95% CI)	*χ*^2^	*p* value	Coefficient	sHR (95% CI)	*χ*^2^	*p* value
Age 60 years or above, per 1 year increase	0.055	1.06 (1.03–1.09)	14.1	< 0.001	0.044	1.05 (1.01–1.08)	8.5	0.004
Male sex								
Heart rate 50–100 beats/min, per 5 beats/min increase								
LVEF 45–60%, per 1% decrease	0.052	1.05 (1.02–1.09)	7.8	0.005				
Diastolic BP up to 80 mmHg, per 1 mmHg decrease	0.028	1.03 (1.00–1.05)	5.2	0.022	0.028	1.03 (1.00–1.06)	4.0	0.044
History of diabetes	0.718	2.05 (1.41–2.98)	14.1	< 0.001	0.758	2.13 (1.45–3.15)	14.5	< 0.001
History of dyslipidemia	− 0.656	0.52 (0.35–0.78)	10.2	0.001	− 0.676	0.51 (0.33–0.79)	9.1	0.003
History of atrial fibrillation	0.588	1.80 (1.23–2.63)	9.3	0.002				
Potassium 4–5.5 mmol/l, per 1 mmol/l increase	0.486	1.63 (1.06–2.50)	4.9	0.027				
Albumin 35–45 g/l, per 1 g/l decrease	0.096	1.10 (1.03–1.18)	7.6	0.006				
Serum creatinine 0.8–2.5 mg/dl, per 0.1 mg/dl increase	0.069	1.07 (1.02–1.12)	8.5	0.004	0.060	1.06 (1.01–1.11)	6.1	0.014
NT-proBNP up to 3000 pg/ml, per 100 pg/ml					0.059	1.06 (1.04–1.08)	31.4	< 0.001

*sHR* subdistribution hazard ratio, *CI* confidence interval, *LVEF* left-ventricular ejection fraction, *BP* blood pressure, *NT-proBNP* N-terminal pro-B-type natriuretic peptide

*χ*^2^ score: the larger *χ*^2^ value, the more powerful the predictor

For the continuous variables, there was no further trend in the risk of pump failure death with age for values < 60 years, for LVEF with values of > 60%, for diastolic blood pressure with values of > 80 mmHg, for potassium with values of < 4 or > 5.5 mmol/l, for albumin with values of < 35 or > 45 g/l, for creatinine with values of < 0.8 or > 2.5 mg/dl, and for NT-proBNP with values of > 3000 pg/ml (Online Fig. A2).

### Performance of the derived models

**Fig. 1:**
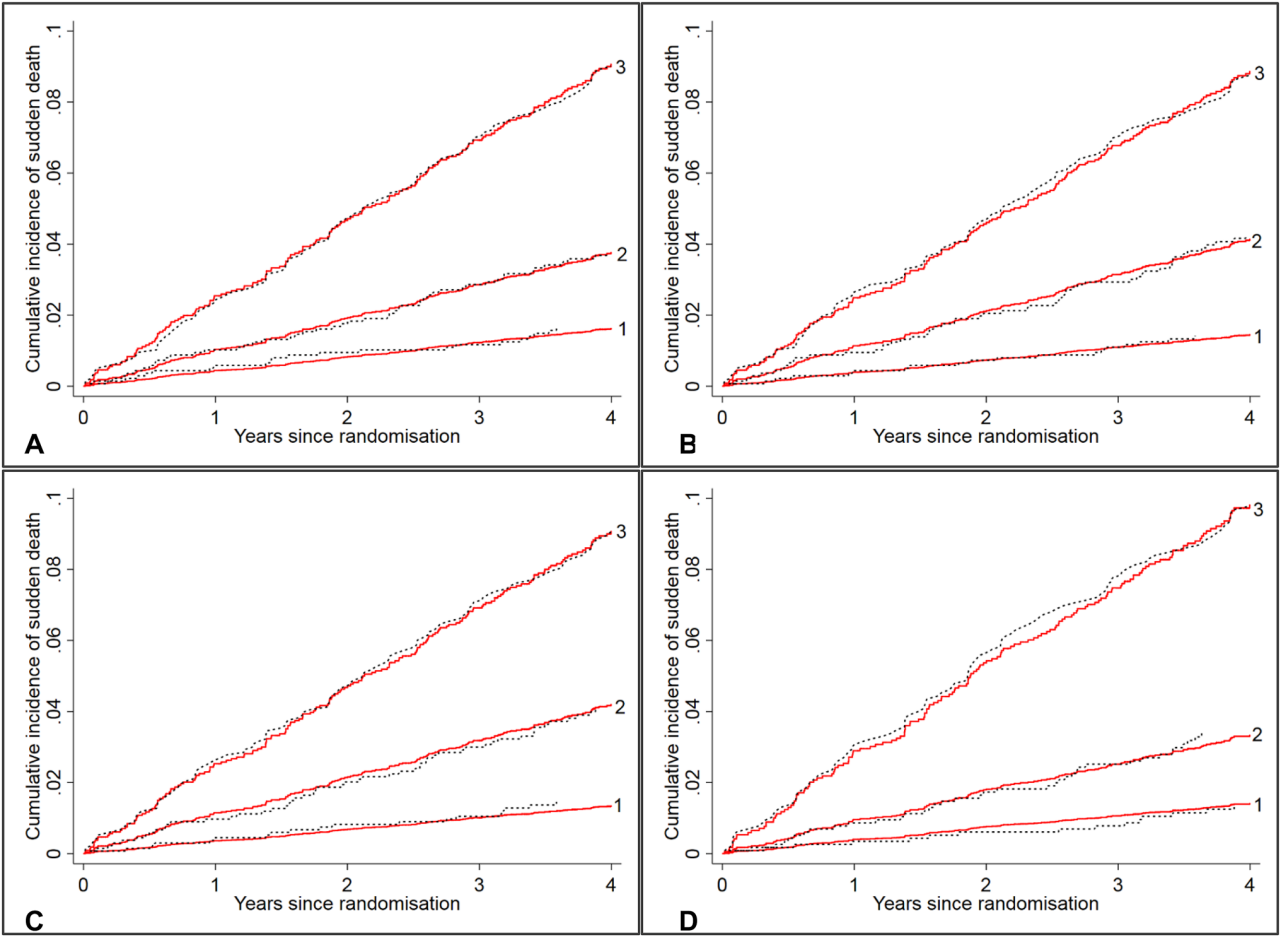
Observed vs. predicted cumulative incidence curves for sudden death by tertile of the risk scores based on the sudden death models in I-Preserve. **a** Sudden death model 1, **b** sudden death model 2, **c** sudden death model 3, **d** sudden death model 4. Red solid lines are predicted cumulative incidence curves based on the corresponding models, and black dotted lines are the observed cumulative incidence curves based on Aalen–Johansen estimators

As can be seen from Fig. 1, each sudden death model showed excellent calibration: the predicted cumulative incidence curve was in good agreement with the corresponding observed one based on the Aalen–Johansen estimator in each tertile of the risk score over time. Both sets of three curves were well separated, suggesting good discrimination; in particular, Model 4 identified the highest tertile with seven times the risk of the lowest tertile. The discrimination was further quantified by the Harrell’s C statistic with values of 0.71 (95% CI 0.68–0.75) in Model 1, 0.72 (95% CI 0.69–0.75) in Model 2, 0.71 (95% CI 0.68–0.75) in Model 3, and 0.75 (95% CI 0.72–0.78) in Model 4.

**Fig. 2:**
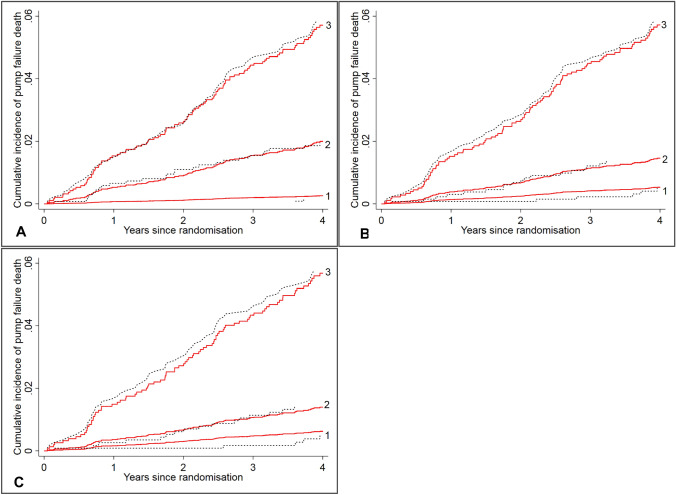
Observed vs. predicted cumulative incidence curves for pump failure death by tertile of the risk scores based on the pump failure death models in I-Preserve. **a** Pump failure death model 1 or 2; **b** pump failure death model 3; **c** pump failure death model 4. Red solid lines are predicted cumulative incidence curves based on the corresponding models, and black dotted lines are the observed cumulative incidence curves based on Aalen–Johansen estimators

For each of the pump failure death models, the predicted and observed cumulative incidences were almost identical in each tertile of the risk score over time, indicating good calibration (Fig. 2). Compared to the lowest tertile, the risk for pump failure death was 12 times higher in the highest tertile in Model 1, and this figure was 13 in Model 3 and 20 in Model 4. The excellent discrimination was confirmed by the Harrell’s C statistic with values of 0.78 (95% CI 0.75–0.82) in Model 1 (or Model 2), 0.80 (95% CI 0.76–0.83) in Model 3, and 0.80 (95% CI 0.76–0.84) in Model 4.

Some violation of proportional subdistribution hazard assumption was observed for creatinine (*p* = 0.043) in the pump failure death models. When graphically displaying the cumulative incidences of pump failure death by tertile of creatinine, the curves generally did not cross over time, suggesting that this was statistically significant but not relevant to the performance of the model (Online Fig. A3).

### External validation of the models in CHARM-Preserved

External validation was performed in 2556 patients in CHARM-Preserved after excluding 23 patients with an ICD or CRT-D at baseline and 444 patients with an LVEF < 45%. There was some difference in baseline characteristics between CHARM-Preserved and I-Preserve. Patients in CHARM-Preserved tended to be younger and more often men with a lower mean LVEF, and were more likely to have an ischemic etiology and a history of myocardial infarction, but had a lower prevalence of previous HF hospitalization and ECG LVH (Table 1).

During a median 36.6 months of follow-up, 409 death events were recorded in CHARM-Preserved, including 110 sudden deaths and 82 pump failure deaths, with the corresponding annual rates of 1.5 (95% CI 1.2–1.8) and 1.1 (95% CI 0.9–1.4) per 100 patient-years, respectively.

For the sudden death models, there was a marginal decrease in discrimination ability when validated in CHARM-Preserved, with a Harrell’s C statistic of 0.68 (95% CI 0.64–0.73) for Model 1 and 0.69 (95% CI 0.65–0.74) for Model 2. For Model 1, the predicted and observed cumulative incidences were broadly similar across tertiles and both sets of curves were evenly distributed. However, Model 2 was less able to discriminate the higher two tertiles and slightly under-predicted the highest tertile but over-estimated the middle tertile in the middle period of follow-up (Online Fig. A4).

For the pump failure death Model 1 (or Model 2), history of dyslipidemia was not recorded and treatment with lipid lowering agents was used instead. Discrimination remained excellent in CHARM-Preserved with a Harrell’s C of 0.79 (95% CI 0.75–0.83), and calibration was generally reasonable over time (Online Fig. A5).

### External validation of the models in TOPCAT

Models developed in I-Preserve were also validated in 3401 patients in TOPCAT, after excluding 44 patients with an ICD or CRT-D. Baseline characteristics were broadly similar between I-Preserve and TOPCAT, but some differences were noted. Compared to those in I-Preserve, patients in TOPCAT were slightly younger and more often men, and had a lower level of mean LVEF, blood pressure, and serum albumin, but had a substantially higher level of median NT-proBNP among the 615 patients (18%) with NT-proBNP available; patients in TOPCAT had a higher average BMI and were more likely to have dyslipidemia, renal dysfunction, or prior HF hospitalization, but had a lower prevalence of ECG LVH (Table 1).

There were 520 death events in TOPCAT over a median 41.1 months of follow-up, including 110 sudden deaths and 65 pump failure deaths with the corresponding annual rates of 1.0 (95% CI 0.8–1.2) and 0.6 (95% CI 0.4–0.7) per 100 patient-years, respectively.

For the sudden death models, a modest decrease in discrimination was observed when validated in TOPCAT, with a Harrell’s C of 0.66 (95% CI 0.61–0.71) for Model 1, 0.65 (95% CI 0.60–0.70) for Model 2, 0.64 (95% CI 0.59–0.69) for Model 3, and 0.73 (95% CI 0.64–0.83) for Model 4, respectively. Despite some disagreement in the middle period of follow-up, the observed and predicted cumulative incidences were generally similar across subgroups except for Model 3 which failed to separate the higher two tertiles, i.e., it over-estimated the highest tertile and underestimated the middle one (Online Fig. A6).

For the pump failure death models, discrimination considerably decreased but remained good in TOPCAT with a Harrell’s C of 0.72 (95% CI 0.65–0.79) for Model 1, 0.71 (95% CI 0.63–0.78) for Model 3, and 0.80 (95% CI 0.68–0.92) for Model 4. In general, the calibration was reasonable in these models, except for Model 1 which did not separate the lower two tertiles (Online Fig. A7).

### Predicting an individual’s risk

The multivariable models presented in Tables 2 and 3 from I-Preserve can be used to calculate an individual’s risk score for sudden death and pump failure death, respectively, by adding up the products of the value and its corresponding coefficient of each prediction variable from each model. Based on the obtained risk score, the corresponding cumulative incidence for each mode of death within 4 years can be estimated using the corresponding curves outlined in Online Fig. A8 and Online Fig. A9, which showed the distribution of the risk scores for each mode of death, based on the clinical model (Model 1) and the model with NT-proBNP (Model 4), and its association with the corresponding predicted cumulative incidence by 4 years in I-Preserve, respectively (Examples are given in Online Supplement).

**Fig. 3:**
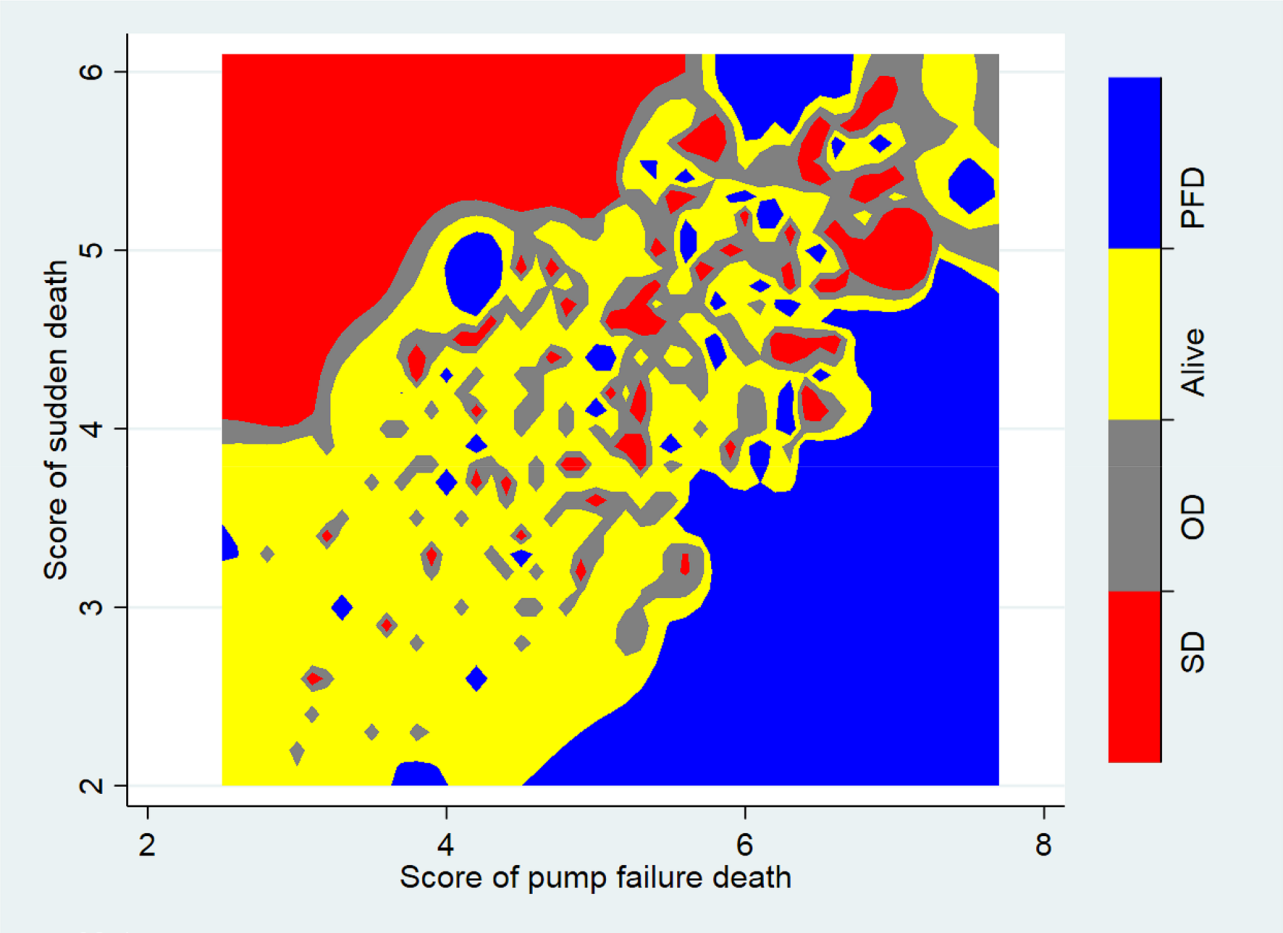
Survival status at the end of follow-up in I-Preserve, according to baseline risk score for sudden death and pump failure death. Every patient has a score for both risk of pump failure death and risk of sudden death at baseline, plotted on the *X* and *Y* axis, respectively. The shaded areas show the outcome for each patient during follow-up, according to their scores (*PFD* pump failure death, *OD* other death, *SD* sudden death). As can be seen, patients who died suddenly (shown in red, clustered in the upper left quadrant of the figure) had a high score for risk of sudden death and low score for risk of pump failure death. The opposite was true for patients dying from pump failure (clustered in the lower right quadrant of the figure)

Figure 3 illustrates the outcome of patients during follow-up in I-Preserve according to their risk scores at baseline for sudden death and pump failure death. As can be seen, these scores clearly identified patients at particular risk of both sudden death and pump failure death, separately (upper left and lower right quadrants, respectively).

### Mode-specific death in the high-risk subgroup based on the sudden death model in I-Preserve and in the control group of SCD-HeFT

**Fig. 4:**
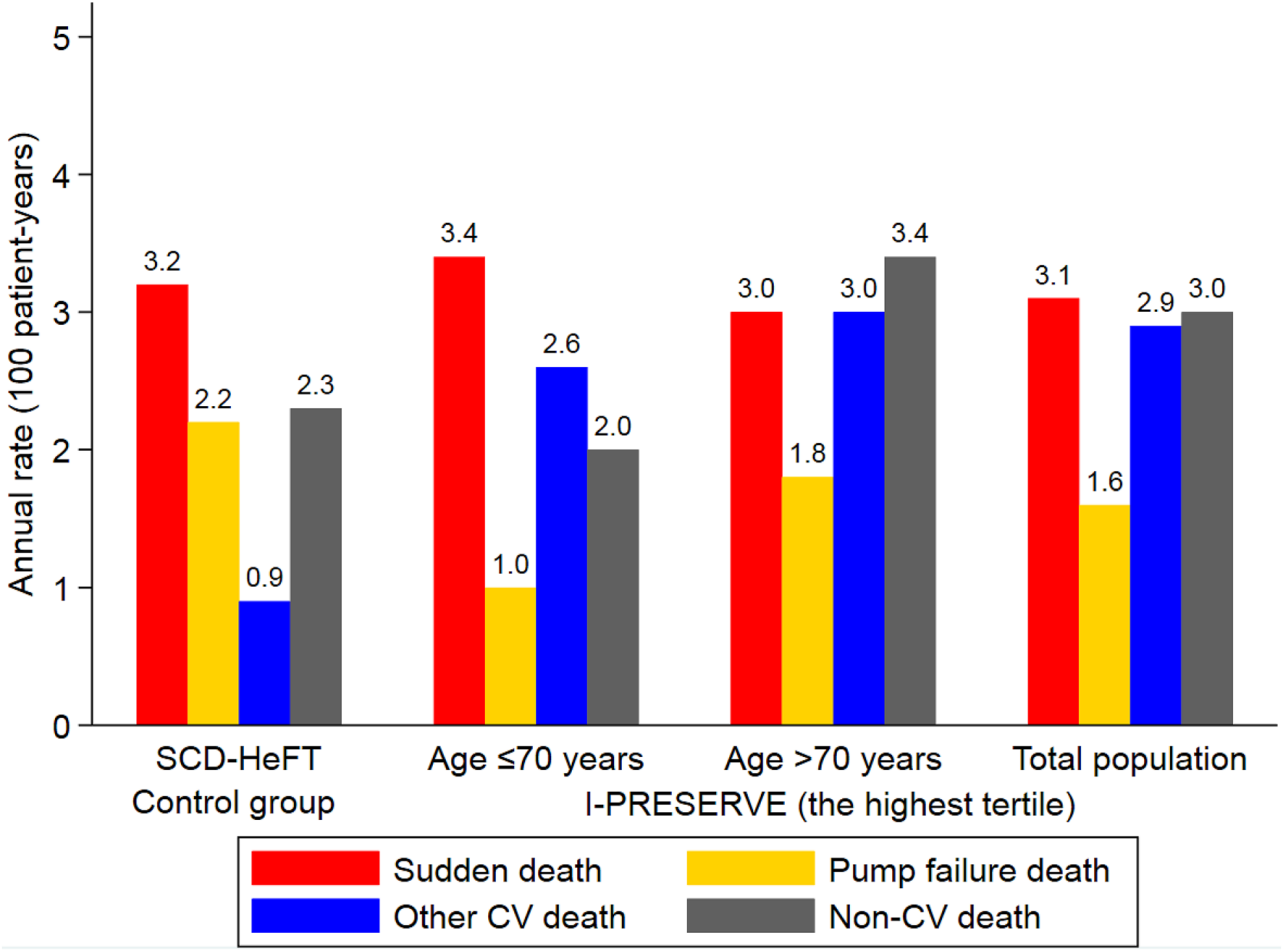
Annual rates of modes of death in the highest tertile based on the sudden death model in I-Preserve and in the control group of SCD-HeFT. The range of the risk score in the highest tertile based on sudden death model 4 in I-Preserve was from 3.8 to 6.1 with the median value of 4.2

The risk highest tertile of patients in I-Preserve (identified using sudden death risk model 4) had an annual rate of sudden death (3.1 per 100 patient-years) similar to that in the control group of SCD-HeFT (3.2 per 100 patient-years), although the former had a slightly higher rate of non-sudden deaths (7.5 vs. 5.4 per 100 patient-years) (Fig. 4). With the addition of age as another stratifier (≤ 70 vs. > 70 years), the highest risk tertile of patients aged ≤ 70 years had nearly identical rates of sudden death and non-sudden deaths as those in the control arm of SCD-HeFT.

## Discussion

Risk stratification for mode-specific death has been studied in patients with HFrEF [14–16], but remains largely unexplored in patients with HFpEF [17–20]. Identification of prognostic factors for mode-specific death may help with the understanding of the pathophysiological mechanisms underlying the cause of death. It may also permit the identification of high-risk subgroups for inclusion in new trials of device interventions or pharmacological therapies, especially as sudden death is a not infrequent occurrence in HFpEF and there is evidence that a high proportion of HFpEF patients with sudden death have a shockable rhythm [20, 21]. This in turn should allow affordable, adequately powered, trials to be conducted in a realistic number of patients who are most likely to benefit from the treatment under investigation.

We developed a series of models to predict sudden death and pump failure death separately in the HFpEF patients enrolled in I-Preserve and externally validated these models in CHARM-Preserved and TOPCAT. We used a broad spectrum of variables which were included in a stepwise manner. The simplest derived (Model 1 using just demographic and clinical variables) showed good calibration and discrimination, especially for pump failure death, and remained robust, despite a modest decrease in discrimination when validated in TOPCAT, possibly because this trial had the lowest rate of sudden death of all three. Interestingly, further integration of ECG parameters alone (Model 2), even in combination with routine laboratory measurements (Model 3), led to little improvement in model performance in the derivation cohort, and led to a decrease in discrimination and calibration (particularly using Model 3) in the validation cohorts. By contrast, the inclusion of NT-proBNP (Model 4) substantially increased the discriminative ability for sudden death and simplified the model for pump failure death, with a marginal increase in discrimination. Both models remained robust when validated in the subset of patients with NT-proBNP measurements in TOPCAT.

There are only two other published models for predicting sudden death in patients with HFpEF. One was also developed in I-Preserve [6]. However, that earlier model was developed using conventional Cox regression analysis rather than using competing risk analysis. Deaths from non-sudden causes were counted as independent censorings, i.e., those individuals were still considered to be at risk for dying suddenly, which was untrue, and over-estimated the cumulative incidence of sudden death, especially in this elderly population with a heavy burden of comorbidities in which death from other causes is frequent [22]. Additionally, that model was not externally validated. Similar considerations apply to a model developed in a subset of patients from TOPCAT in which there were only 23 sudden deaths [19]. We tried to rectify these limitations in the present study, especially with a view to considering a potential role for primary prevention ICD therapy in this heart failure phenotype. In this context, the competing risk of non-cardiovascular death is of particular concern in HFpEF. Without a favorable ratio of sudden to non-sudden deaths (including non-cardiovascular deaths), ICD therapy is unlikely to reduce all-cause mortality or to be affordable. SCD-HeFT remains the only large trial demonstrating an overall survival benefit of primary prevention ICD therapy in patients with HFrEF, but patients enrolled in that trial were more than a decade younger than the patients in I-Preserve (60 vs. 72 years respectively) [7, 13]. With increasing age, and associated comorbidity, there is a shift towards non-cardiovascular causes of death in both HFrEF and HFpEF, and, consequently, the potential survival benefit from ICD therapy is reduced [23]. This was shown in a recent analysis of the Danish Study to Assess the Efficacy of ICDs in Patients with Non-Ischemic Systolic Heart Failure on Mortality (DANISH), where the survival benefit of ICDs decreased linearly with age. The optimal age cut-off age in DANISH was ≤ 70 years [24]. When we used age ≤ 70 years as an additional stratifier in the present study, we were able to identify a subgroup of patients with the rates of sudden death and non-sudden deaths, including non-cardiovascular deaths, similar to those in SCD-HeFT [13]. Therefore, it is possible that ICD therapy could reduce mortality in this substantial subgroup of patients with HFpEF, although this hypothesis needs to be tested in a randomized clinical trial.

Although the focus has been on sudden death, we also looked at the risk of death from pump failure. There is no existing predictive model for this mode of death in HFpEF. The series of models we developed to predict pump failure death included variables known to predict death and HF hospitalization, such as age, heart rate, LVEF, history of diabetes or atrial fibrillation, creatinine, and NT-proBNP [25]. Interestingly, prior HF hospitalization, which had been reported as a strong predictor of HF hospitalization and death [25, 26], was not selected in our models for pump failure death (but was included in the sudden death models). The reason for this possibly surprising finding is uncertain. One explanation is that patients with mild symptoms could only be included in I-Preserve if they had an HF hospitalization within the previous 6 months and patients with mild symptoms are at higher risk of sudden death than pump failure death (at least in HFrEF) [7]. Another interesting observation was the association between dyslipidaemia and lower risk of pump failure death. While this might reflect the play of chance, cholesterol levels decrease with increasing heart failure severity, and in keeping with this, the median level of NT-proBNP was considerably lower in patients with dyslipidaemia compared to those without (287 vs. 386 pg/ml, respectively) [27].

Our study has some limitations. First, validation of the models with NT-proBNP was performed in a relatively small subset of patients (*N* = 615) from TOPCAT and the distribution of NT-proBNP levels differed between the two cohorts. Second, in TOPCAT, there was a substantial variation in the baseline characteristics and clinical outcomes between regions of enrolment (Americas vs. Russia and Georgia). However, in a sensitivity analysis, validating the models in the Americas subgroup, the sudden death model was robust, although the pump failure model showed a decrease in discrimination, possibly due to small sample size and number of events (data not shown) [28]. Third, patient characteristics varied between trials, especially NYHA class, which was more advanced in I-Preserve than the other two trials, probably because of the specific inclusion criteria in I-Preserve. Fourth, these models were developed and tested in clinical trial cohorts which tend to be healthier than “real-world” patients. While it is also important to test the performance of the models in real-world cohorts, it is in patients similar to those in trials that device therapies are most likely to be considered. Fifth, in line with models in HFrEF, sudden death models were less discriminative than pump failure death models [14, 15]. This suggests that there is a room to improve the prediction of sudden death in heart failure. Late gadolinium enhancement (LGE) on cardiovascular magnetic resonance (CMR) imaging appears to be a promising variable in this respect in HFrEF, but its value in HFpEF is unknown [29]. Finally, the vast majority of patients in all cohorts were Caucasian and, ideally, our models should be re-validated in more racially diverse cohorts.

In conclusion, prognostic models developed using simple demographic and clinical variables predicted the risks of sudden death and pump failure death, separately, in patients with HFpEF, with good discrimination and calibration, and were robust in external validation. Inclusion of NT-proBNP further improved the performance of both models. These models may have important clinical implications for identifying high-risk patients for specific interventions in future trials among patients with HFpEF.

## Supplementary Information

Below is the link to the electronic supplementary material.

Supplementary file1 (DOCX 3096 KB)

## Data Availability

The data used in this study are not available to others. The trial sponsors (AstraZeneca for CHARM-Preserved and Bristol-Myers Squibb and Sanofi-Aventis for I-preserve) are committed to sharing with qualified external researchers, access to patient-level data, and supporting clinical documents from eligible studies. Access to TOPCAT is open for application from BioLINCC (https://biolincc.nhlbi.nih.gov/home/).

## Code availability

STATA.
