# Supplementary material for: Developing and validating models to predict sudden death and pump failure death in patients with heart failure and preserved ejection fraction
Source: Clin Res Cardiol. 2020 Dec 10;110(8):1234–48. doi: 10.1007/s00392-020-01786-8 (PMC8318942; doi:10.1007/s00392-020-01786-8)
Supplement: Supplementary file 1 — Supplementary file1 (DOCX 3096 KB) [file 392_2020_1786_MOESM1_ESM.docx]

ONLINE SUPPLEMENTAL MATERIAL

**Online Tables**

**Online Table 1** Definitions of sudden death and pump failure death used in the included trials

Online Table 2 The 25 most powerful predictors for sudden death based on univariate analysis in I-Preserve

Online Table 3 The 25 most powerful predictors for pump failure death based on univariate analysis in I-Preserve

**Online Figures**

Online Fig. A1 Histograms of age (A), LVEF (B), serum albumin (C), and NT-proBNP (D) and the corresponding spline curves with the risk of sudden death in I-Preserve

Online Fig. A2 Histograms of age (A), LVEF (B), diastolic blood pressure (C), potassium (D), albumin (E), creatinine (F) and NT-proBNP (G) and the corresponding spline curves with the risk of pump failure death in I-Preserve

**Online Fig. A3** Cumulative incidence curves for pump failure death by tertile of serum creatinine level in I-Preserve

Online Fig. A4 Observed vs. predicted cumulative incidence curves for sudden death by tertile of the risk scores in CHARM-Preserved

Online Fig. A5 Observed vs. predicted cumulative incidence curves for pump failure death by tertile of the risk scores in CHARM-Preserved

Online Fig. A6 Observed vs. predicted cumulative incidence curves for sudden death by subgroup of the risk scores in TOPCAT

Online Fig. A7 Observed vs. predicted cumulative incidence curves for pump failure death by subgroup of the risk scores in TOPCAT

Online Fig. A8 Distribution of risk score for sudden death and its relation to cumulative incidence of sudden death within 4 years in I-Preserve

Online Fig. A9 Distribution of risk score for pump failure death and its relation to cumulative incidence of pump failure death within 4 years in I-Preserve

**Online supplement:** Examples of risk prediction for mode-specific death using the model 4 presented in Table 2 and 3

Online Table 1 Definitions of sudden death and pump failure death used in the included trials

| **Trial** | **Definition of sudden death** | **Definition of pump failure death** |
| --- | --- | --- |
| I-Preserve | Unexpected death in a previously stable patient. This includes patients who were comatose then died after attempted resuscitation. Patients in this category should have had recent human contact before the event. Patients who die who have been out of contact for prolonged or unknown periods of time will be classified as unknown. | Death from worsening/intractable HF which generally occur during hospitalization but can occur at home during hospice care. Terminal arrhythmias associated with pump failure deaths will be classified as a pump failure death. Pump failure secondary to a recent myocardial infarction will be classified as an MI death. |
| CHARM-Preserved | Death that occurred unexpectedly in an otherwise stable patient. Examples may include: the time of death is unknown; for identified arrhythmic death; in the absence of medical care; patient is unable to be resuscitated from cardiac arrest; and patient who later dies from an attempted resuscitation. | Death occurring within the context of clinically worsening symptoms and/or signs of HF without evidence of another cause of death. If worsening HF is secondary to MI, then MI should be listed as the primary cause of death given that the patient suffered a MI within 14 days of death. |
| TOPCAT | Death that occurred unexpectedly in an otherwise stable subject. Further sub-classification of sudden death will be as follows: witnessed ***or*** last seen ≥1 and <24 hours. | Same as the definition of pump failure death in CHARM-Preserved. |

Online Table 2 The 25 most powerful predictors for sudden death based on univariate analysis in I-Preserve

| **Variable** | **sHR (95% CI)** | **p value** | **X^2^ score** |
| --- | --- | --- | --- |
| NT-proBNP up to 3000 pg/ml, per 100 pg/ml increase | 1.06 (1.05-1.08) | <0.001 | 96.0 |
| LVEF 45-60%, per 1% decrease | 1.08 (1.05-1.10) | <0.001 | 34.0 |
| Blood urea nitrogen 15-55 mg/dl, per 1 mg/dl increase | 1.03 (1.02-1.05) | <0.001 | 29.6 |
| Male sex | 1.92 (1.48-2.49) | <0.001 | 24.1 |
| Serum creatinine 0.8-2.5 mg/dl, per 0.1 mg/dl increase | 1.09 (1.05-1.13) | <0.001 | 22.9 |
| Ischemic aetiology | 1.89 (1.45-2.47) | <0.001 | 22.2 |
| Age 60 years or above, per 1 year increase | 1.05 (1.03-1.07) | <0.001 | 22.1 |
| QRS duration 90-130 msec, per 5 msec increase | 1.10 (1.05-1.14) | <0.001 | 20.3 |
| History of myocardial infarction | 1.82 (1.39-2.38) | <0.001 | 18.7 |
| Albumin 35-45 g/L, per 1 g/L decrease | 1.10 (1.05-1.15) | <0.001 | 15.1 |
| Neutrophil -10^9^/L, per 10^9^/L increase | 1.09 (1.04-1.14) | <0.001 | 14.5 |
| Leukocyte 6-10*10^9^/L, per 10^9^/L increase | 1.18 (1.08-1.29) | <0.001 | 13.3 |
| History of diabetes | 1.64 (1.26-2.14) | <0.001 | 13.2 |
| History of COPD or asthma | 1.91 (1.34-2.72) | <0.001 | 13.0 |
| HF hospitalization within previous 6 months | 1.60 (1.23-2.07) | <0.001 | 12.5 |
| Left bundle branch block on ECG | 1.84 (1.27-2.68) | 0.001 | 10.2 |
| Bundle branch block on ECG | 1.61 (1.18-2.21) | 0.003 | 8.9 |
| BMI 18-45 kg/m^2^, per 1 kg/m^2^ increase | 0.96 (0.93-0.99) | 0.004 | 8.5 |
| eGFR up to 60 ml/min/1.73m^2^, per 1 ml/min/1.73m^2^ increase | 0.98 (0.96-0.99) | 0.004 | 8.5 |
| Heart rate 50-100 beats/min, per 5 beats/min increase | 1.09 (1.03-1.16) | 0.004 | 8.1 |
| History of atrial fibrillation | 1.46 (1.11-1.90) | 0.006 | 7.6 |
| Potassium 4-6 mmol/L, per 1 mmol/L increase | 1.49 (1.11-2.00) | 0.008 | 6.9 |
| Left ventricular hypertrophy on ECG | 1.29 (0.99-1.69) | 0.061 | 3.5 |
| Atrial fibrillation or flutter on ECG | 1.33 (0.97-1.83) | 0.076 | 3.2 |
| History of dyslipidemia | 0.80 (0.61-1.04) | 0.097 | 2.8 |

sHR denotes sub-distribution hazard ratio; CI, confidence interval; NT-proBNP, N terminal pro-B type natriuretic peptide; LVEF, left ventricular ejection fraction; COPD, chronic obstructive pulmonary disease; BMI, body mass index; eGFR, estimated glomerular filtration rate.

X^2^ score: the larger X2 value, the more powerful the predictor.

Online Table 3 The 25 most powerful predictors for pump failure death based on univariate analysis in I-Preserve

| **Variable** | **sHR (95% CI)** | **p value** | **X^2^ score** |
| --- | --- | --- | --- |
| NT-proBNP up to 3000 pg/ml, per 100 pg/ml increase | 1.08 (1.06-1.10) | <0.001 | 81.7 |
| Blood urea nitrogen 15-55 mg/dl, per 1mg/dl increase | 1.06 (1.04-1.07) | <0.001 | 62.7 |
| Serum creatinine 0.8-2.5 mg/dl, per 0.1 mg/dl increase | 1.15 (1.11-1.19) | <0.001 | 53.1 |
| Age 60 years or above, per 1 year increase | 1.08 (1.06-1.11) | <0.001 | 41.0 |
| eGFR up to 80 ml/min/1.73m^2^, per 1 ml/min/1.73m^2^ increase | 0.97 (0.96-0.98) | <0.001 | 39.3 |
| eGFR <60 ml/min/1.73m^2^ | 2.79 (1.95-4.00) | <0.001 | 31.4 |
| Atrial fibrillation or flutter on ECG | 2.74 (1.89-3.97) | <0.001 | 28.4 |
| Neutrophil 4-10*10^9^/L, per 10^9^/L increase | 1.34 (1.20-1.50) | <0.001 | 26.3 |
| History of atrial fibrillation | 2.52 (1.77-3.59) | <0.001 | 26.3 |
| Albumin 35-45 g/L, per 1 g/L decrease | 1.16 (1.10-1.24) | <0.001 | 24.1 |
| Diastolic BP up to 80 mmHg, per 1 mmHg decrease | 1.05 (1.03-1.07) | <0.001 | 22.9 |
| Leukocyte 6-13*10^9^/L, per 10^9^/L increase | 1.22 (1.10-1.35) | <0.001 | 14.5 |
| History of diabetes | 2.00 (1.40-2.85) | <0.001 | 14.4 |
| LVEF 45-60%, per 1% decrease | 1.06 (1.03-1.10) | <0.001 | 12.3 |
| HF hospitalization within previous 6 months | 1.86 (1.29-2.66) | 0.001 | 11.4 |
| QRS duration 90-130 msec, per 1 msec increase | 1.02 (1.01-1.03) | 0.001 | 11.0 |
| Potassium 4-5.5 mmol/L, per 1 mmol/L increase | 2.01 (1.31-3.07) | 0.001 | 10.4 |
| History of dyslipidemia | 0.54 (0.37-0.80) | 0.002 | 9.5 |
| BMI up to 30 kg/m^2^, per 1 kg/m^2^ increase | 0.91 (0.85-0.97) | 0.003 | 8.9 |
| Pacemaker use | 2.21 (1.29-3.79) | 0.004 | 8.4 |
| Heart rate 50-100 beats/min, per 5 beats/min increase | 1.13 (1.04-1.23) | 0.004 | 8.1 |
| Ischemic aetiology | 1.69 (1.17-2.45) | 0.005 | 7.8 |
| History of hypertension | 0.56 (0.35-0.88) | 0.012 | 6.4 |
| Male sex | 1.57 (1.10-2.23) | 0.013 | 6.3 |
| Bundle branch block on ECG | 1.70 (1.12-2.60) | 0.014 | 6.1 |

sHR denotes sub-distribution hazard ratio; CI, confidence interval; NT-proBNP, N terminal pro-B type natriuretic peptide; eGFR, estimated glomerular filtration rate; BP, blood pressure; LVEF, left ventricular ejection fraction; HF, heart failure; BMI, body mass index.

X^2^ score: the larger X2 value, the more powerful the predictor.

Online Fig. A1 Histograms of age (A), LVEF (B), serum albumin (C), and NT-proBNP (D) and the corresponding spline curves with the risk of sudden death in I-Preserve


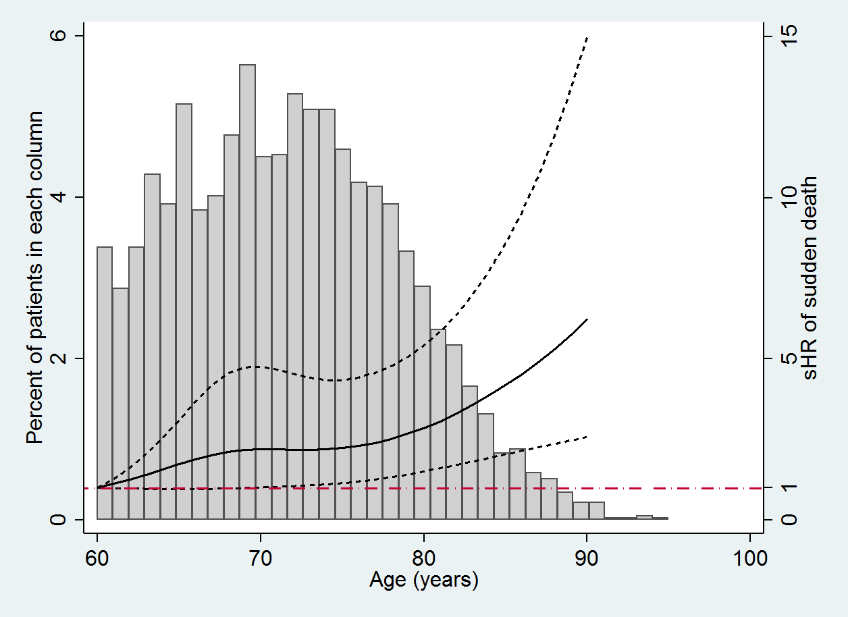

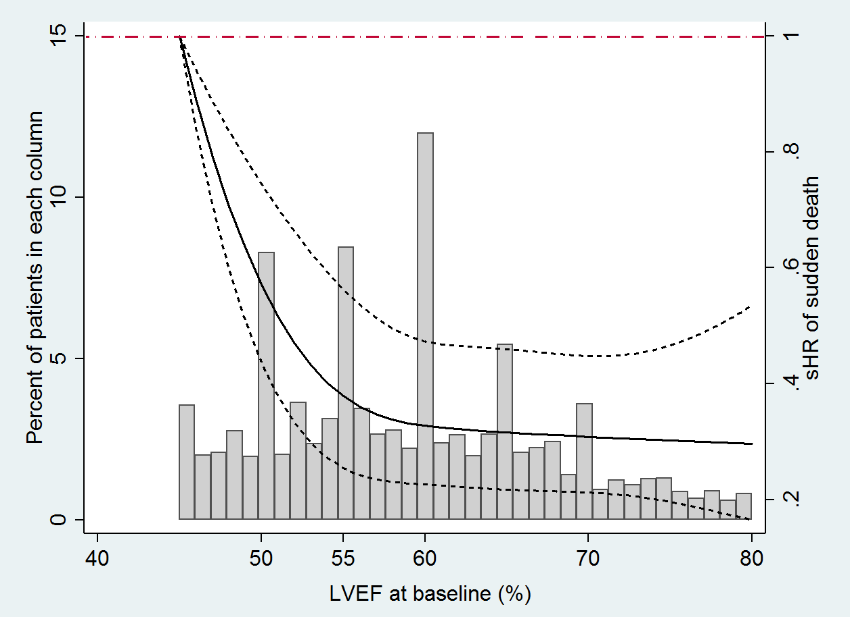

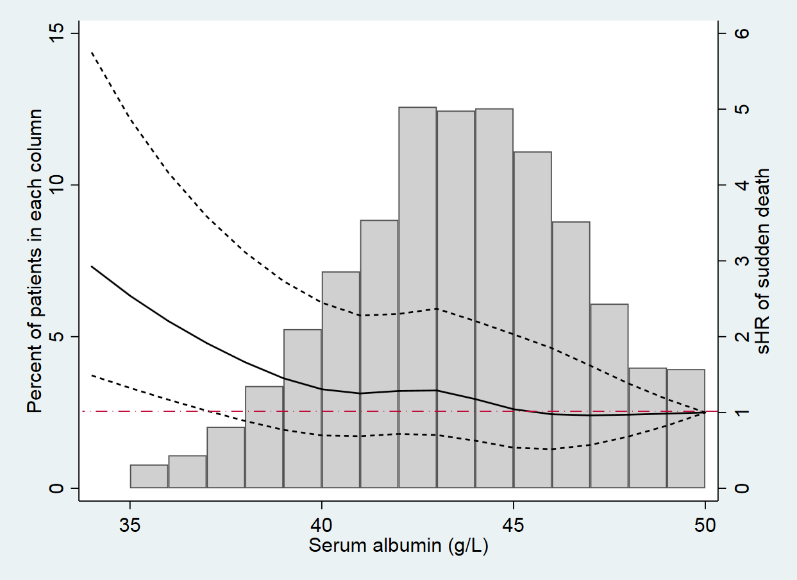

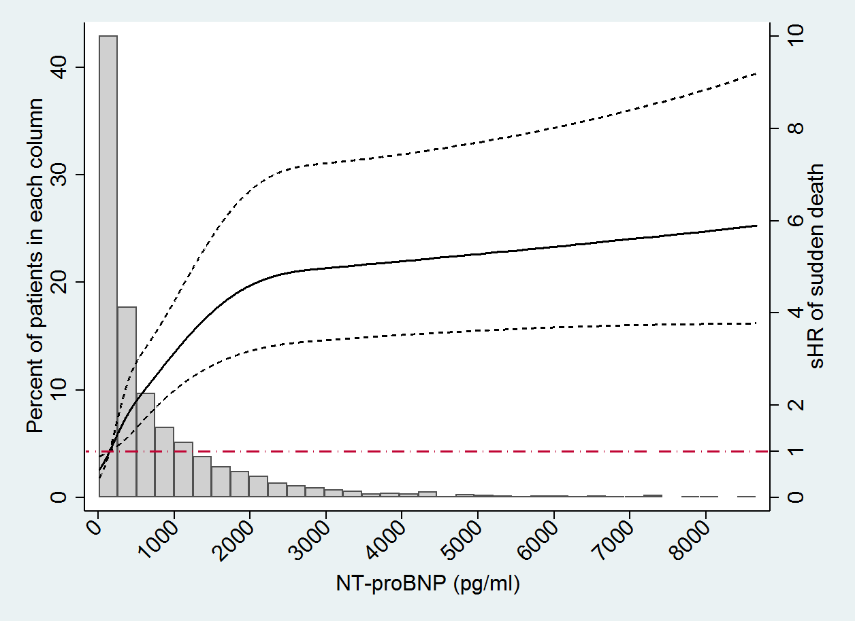


**A**

**B**

**C**

**D**

The columns are the histogram of a continuous variable, and the left axis shows the percent of patients in each column. The black solid line is the sub-distribution hazard ratio (sHR) of sudden death with the corresponding continuous variable and the black dot lines are the 95% confidence intervals. The red dash-dot line is the reference line with a sHR of 1.0 (i.e. no association).

Online Fig. A2 Histograms of age (A), LVEF (B), diastolic blood pressure (C), potassium (D), albumin (E), creatinine (F) and NT-proBNP (G) and the corresponding spline curves with the risk of pump failure death in I-Preserve


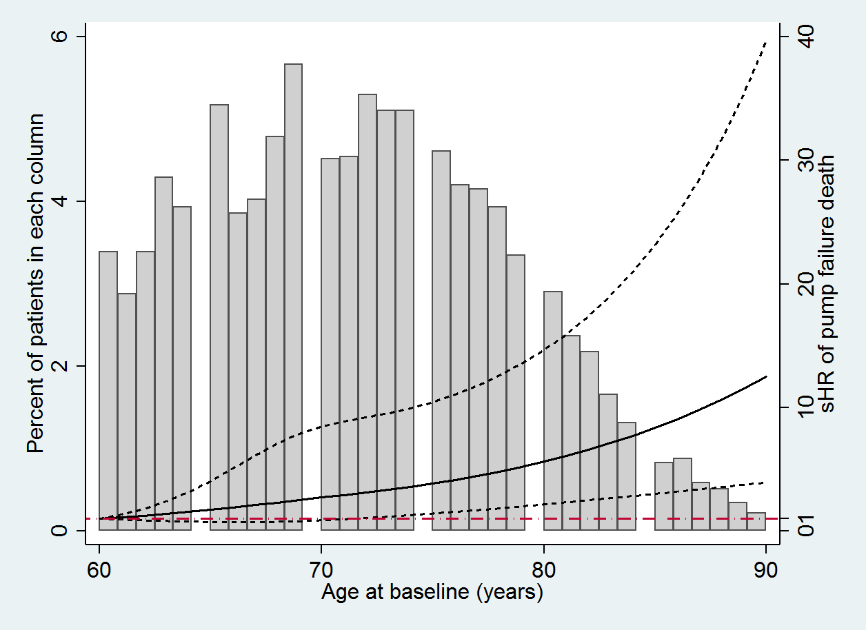

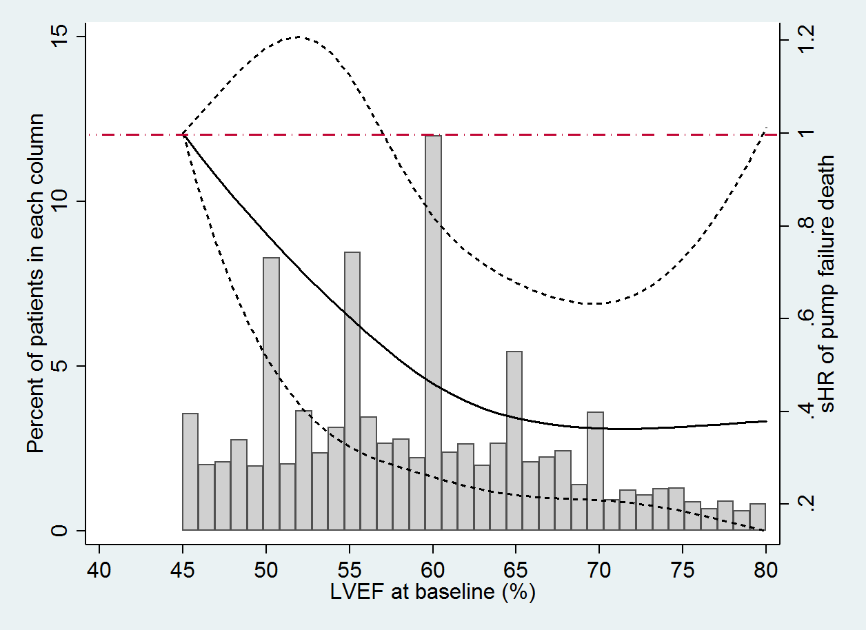

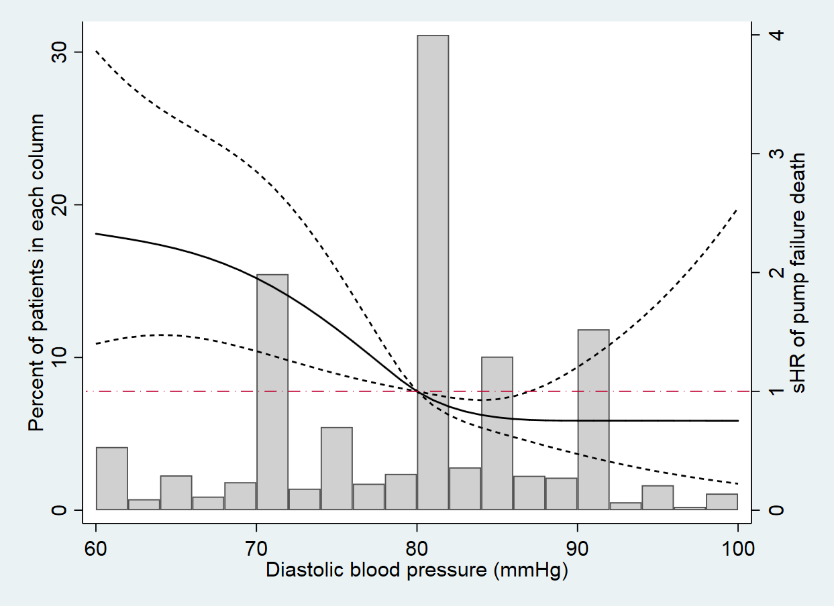

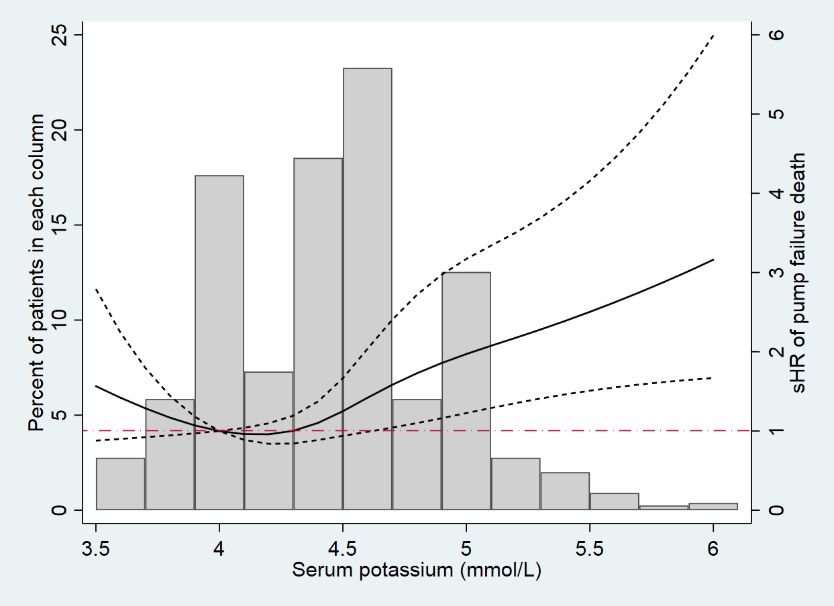

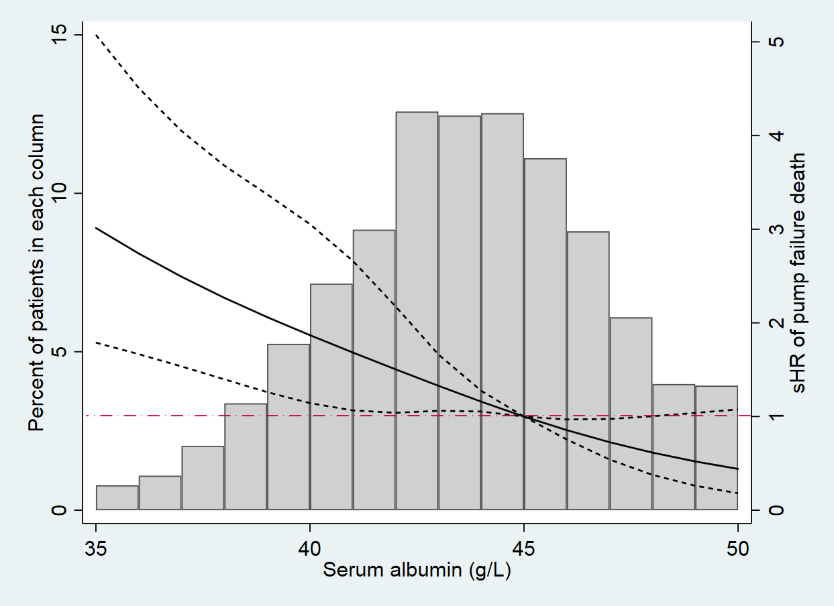

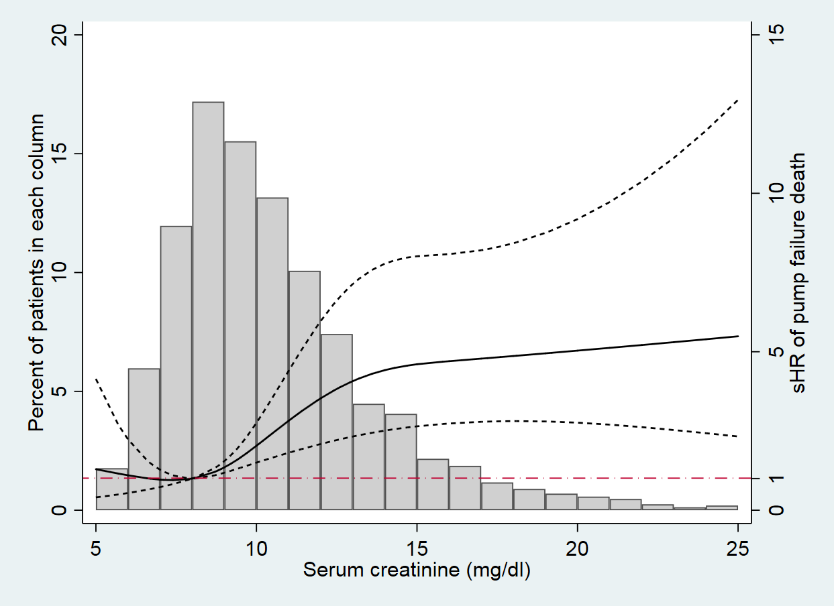

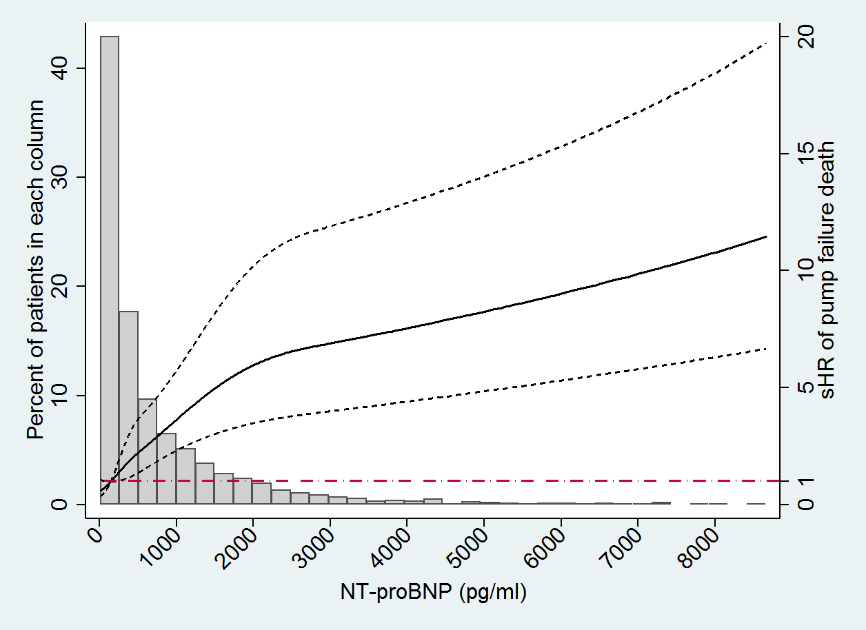


**A**

**B**

**C**

**D**

**E**

**F**

**G**

The columns are the histogram of a continuous variable, and the left axis shows the percent of patients in each column. The black solid line is the sub-distribution hazard ratio (sHR) of pump failure death with the corresponding continuous variable and the black dot lines are the 95% confidence intervals. The red dash-dot line is the reference line with a sHR of 1.0 (i.e. no association).

Online Fig. A3 Cumulative incidence curves for pump failure death by tertile of serum creatinine level in I-Preserve


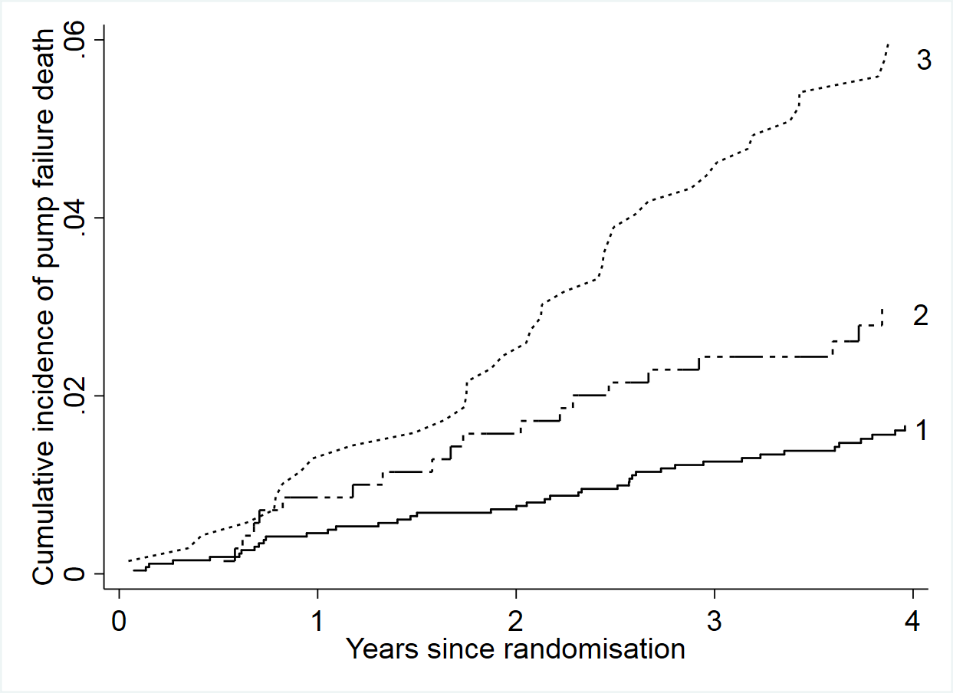


Online Fig. A4 Observed vs. predicted cumulative incidence curves for sudden death by tertile of the risk scores in CHARM-Preserved

**
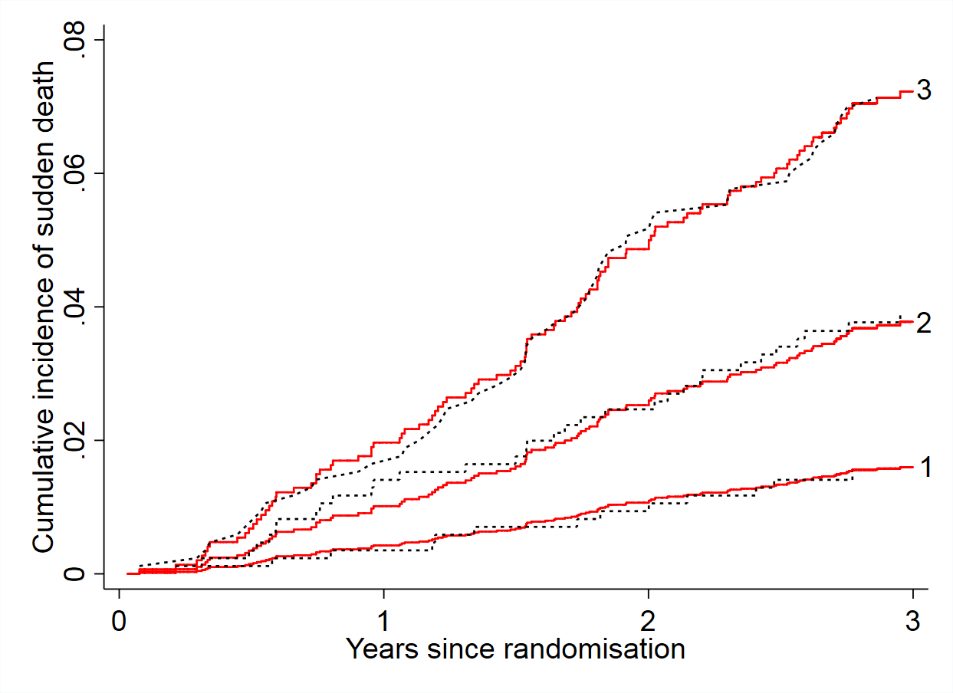

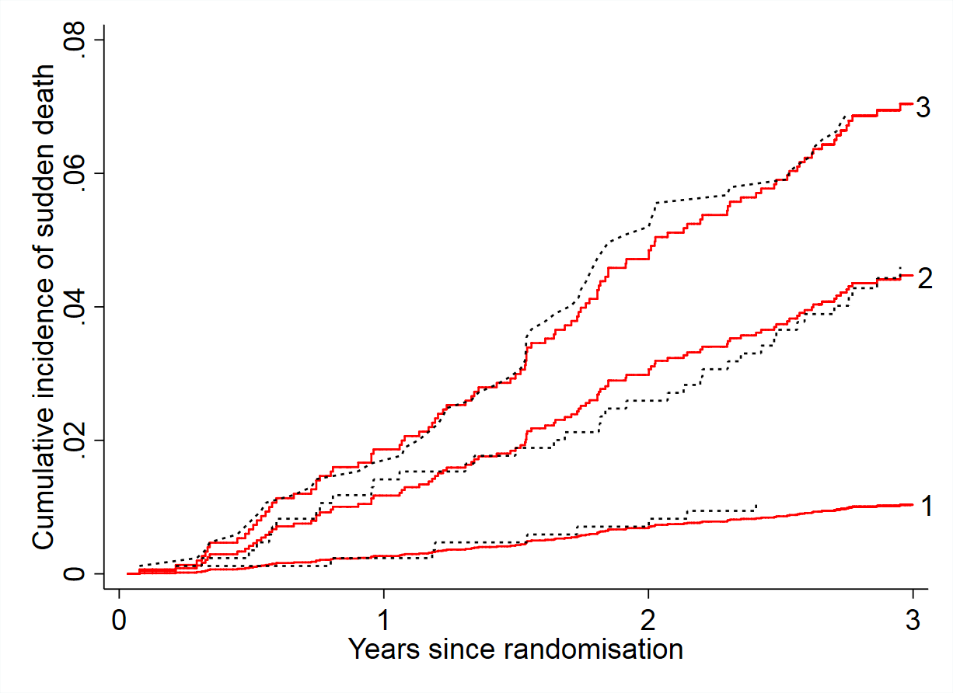
**

**B**

**A**

Panel A. Validation of sudden death model 1 from I-Preserve in CHARM-Preserved; Panel B. Validation of sudden death model 2 from I-Preserve in CHARM-Preserved. Red solid lines are predicted cumulative incidence curves based the corresponding models, and black dotted lines are the observed cumulative incidence curves based on Aalen-Johansen estimators.

Because laboratory measurements were not examined in the majority of patients in CHARM-Preserved, sudden death model 3 and model 4 from I-Preserve were not validated in CHARM-Preserved.

Online Fig. A5 Observed vs. predicted cumulative incidence curves for pump failure death by tertile of the risk scores in CHARM-Preserved


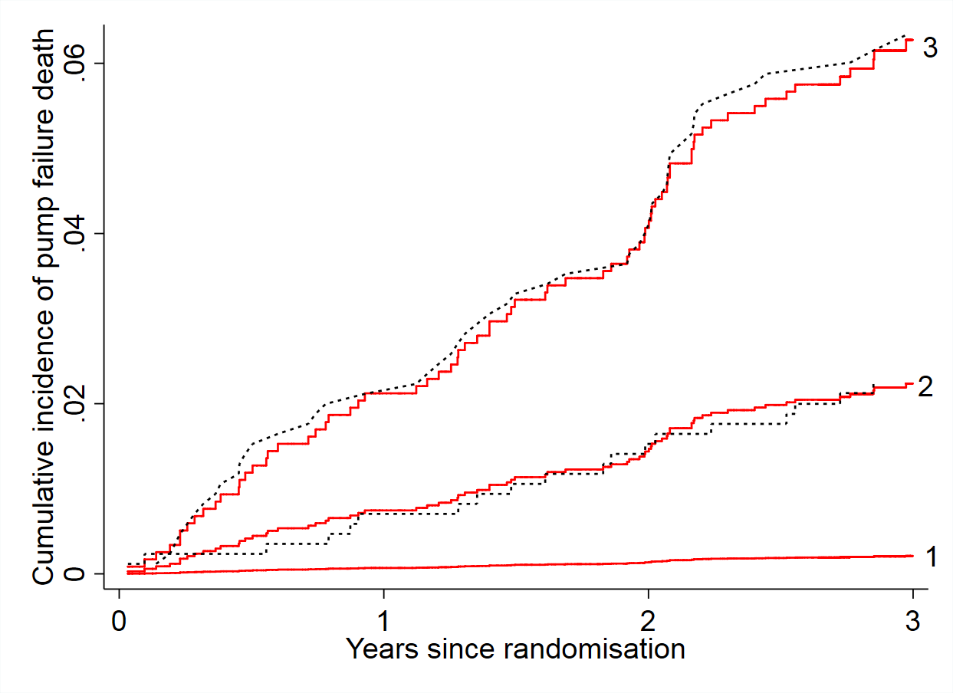


Validation of the pump failure death model 1 (or Model 2) from I-Preserve in CHARM-Preserved. Red solid lines are predicted cumulative incidence curves based the corresponding models, and black dotted lines are the observed cumulative incidence curves based on Aalen-Johansen estimators.

Because laboratory measurements were not examined in the majority of patients in CHARM-Preserved, pump failure death model 3 and model 4 from I-Preserve were not validated in CHARM-Preserved.

Online Fig. A6 Observed vs. predicted cumulative incidence curves for sudden death by subgroup of the risk scores in TOPCAT


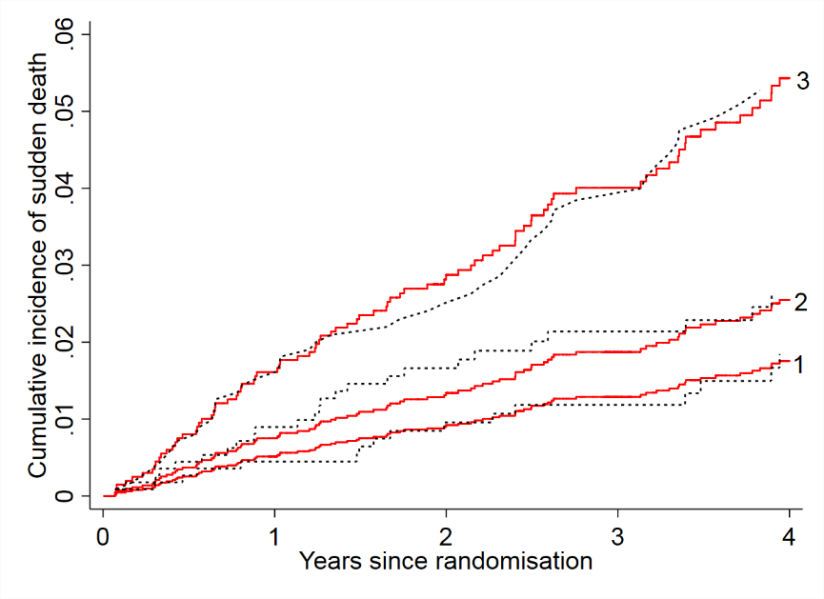

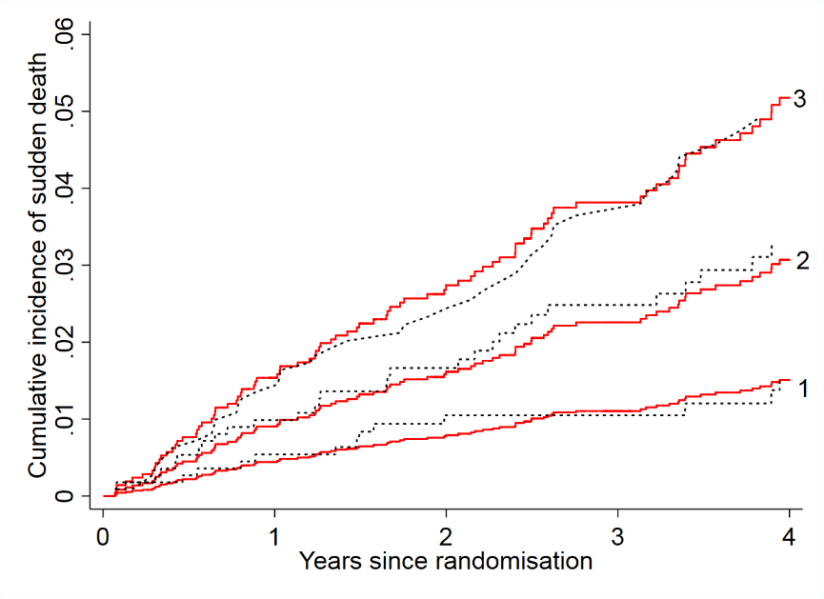

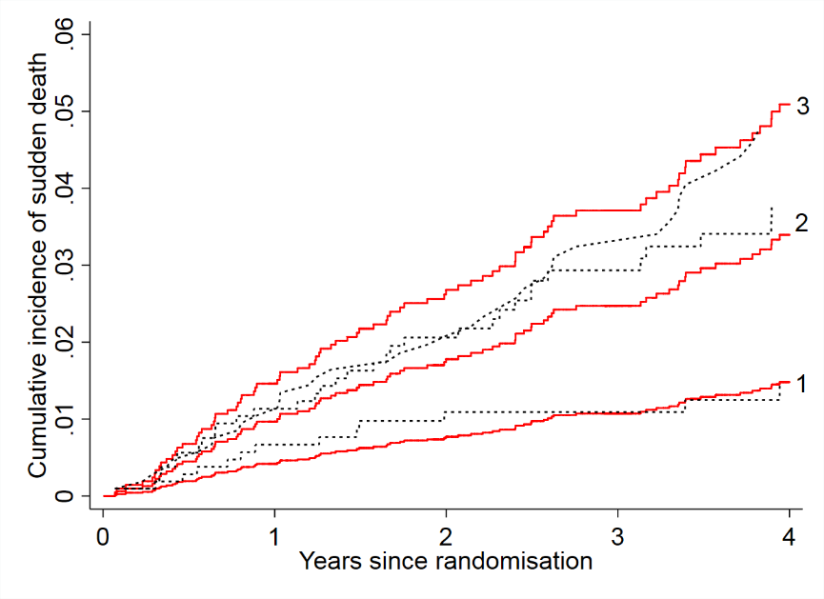

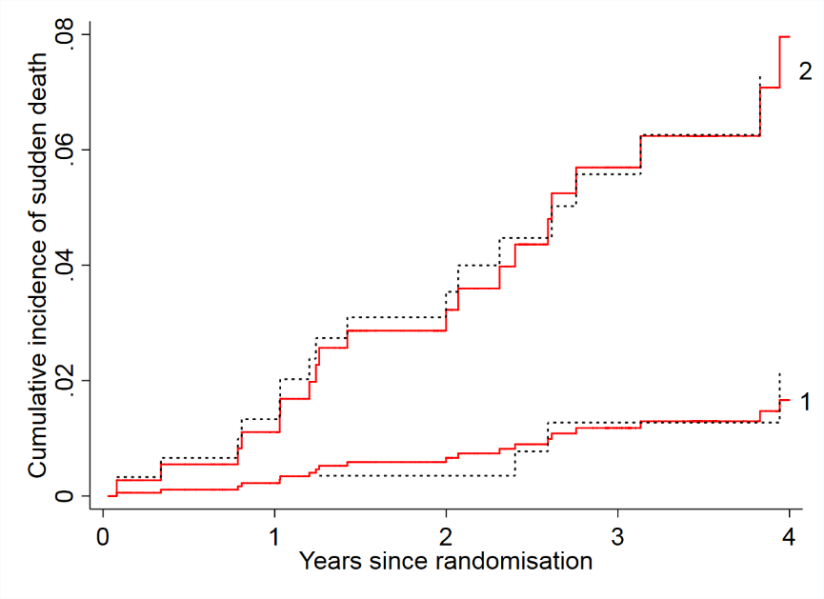


**A**

**C**

**D**

**B**

Panel A. Validation of sudden death model 1 from I-Preserve in TOPCAT; Panel B. Validation of sudden death model 2 from I-Preserve in TOPCAT; Panel C. Validation of sudden death model 3 from I-Preserve in TOPCAT; Panel D. Validation of sudden death model 4 from I-Preserve in TOPCAT.

Red solid lines are predicted cumulative incidence curves based the corresponding models, and black dotted lines are the observed cumulative incidence curves based on Aalen-Johansen estimators.

Given the small cohort size of patients with NT-proBNP measurements in TOPCAT (N=615 [18%]), the risk score of Model 4 was categorised into 2 other than 3 subgroups.

Online Fig. A7 Observed vs. predicted cumulative incidence curves for pump failure death by subgroup of the risk scores in TOPCAT


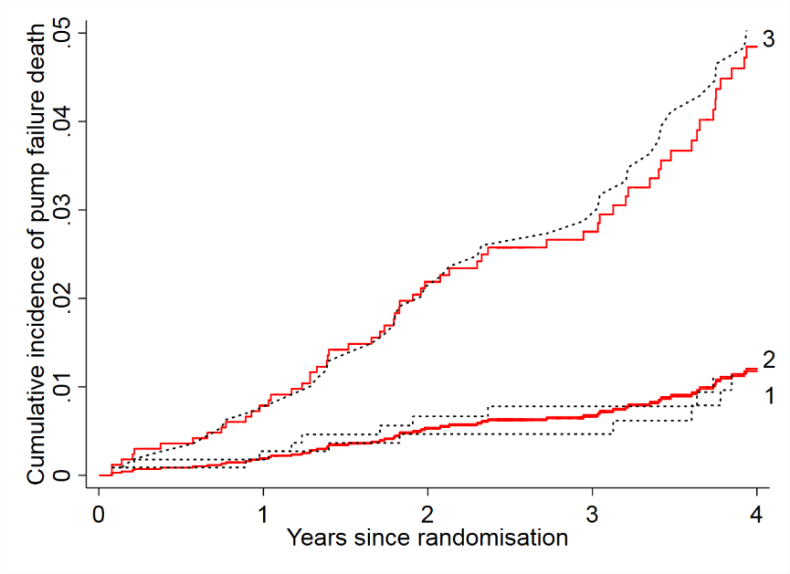

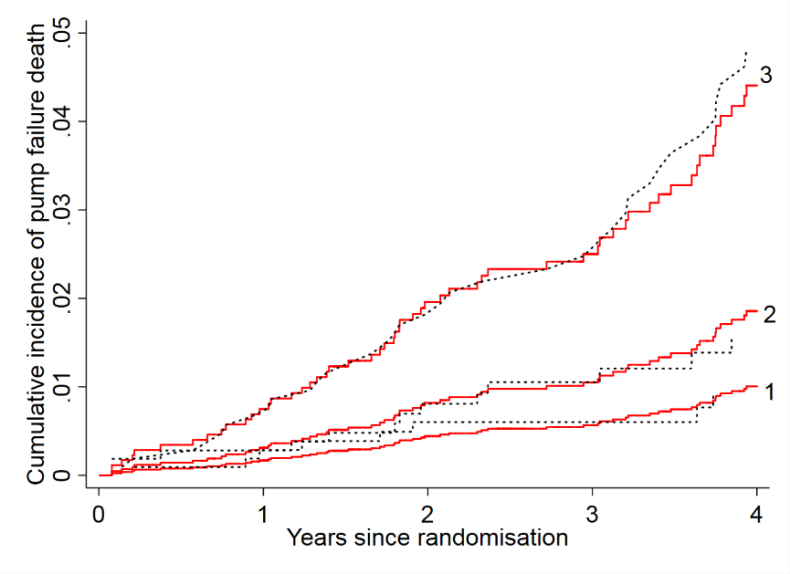

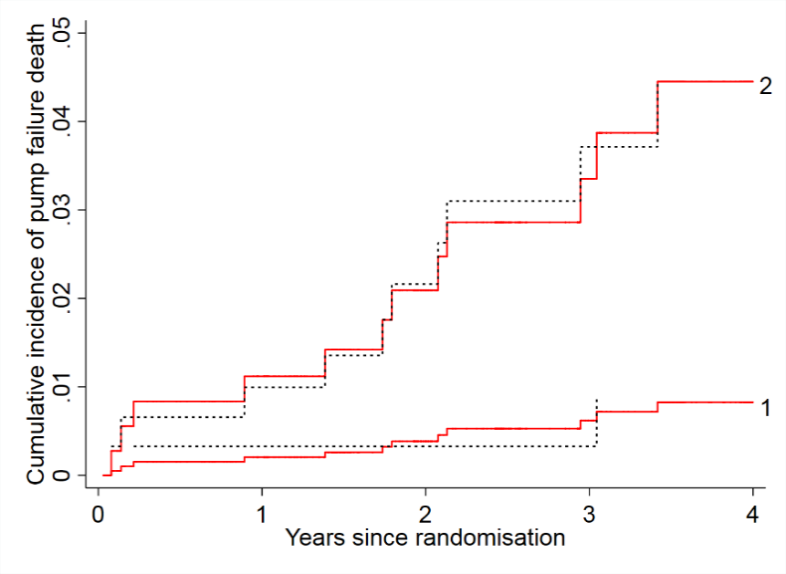


**A**

**B**

**C**

Panel A. Validation of the pump failure death model 1 (or Model 2) from I-Preserve in TOPCAT; Panel B. Validation of the pump failure death model 3 from I-Preserve in TOPCAT; Panel C. Validation of the pump failure death model 4 from I-Preserve in TOPCAT. Red solid lines are predicted cumulative incidence curves based the corresponding models, and black dotted lines are the observed cumulative incidence curves based on Aalen-Johansen estimators. Given the small cohort size of patients with NT-proBNP measurements in TOPCAT (N=615 [18%]), the risk score of Model 4 was categorised into 2 other than 3 subgroups.

Online Fig. A8 Distribution of risk score for sudden death and its relation to cumulative incidence of sudden death within 4 years in I-Preserve


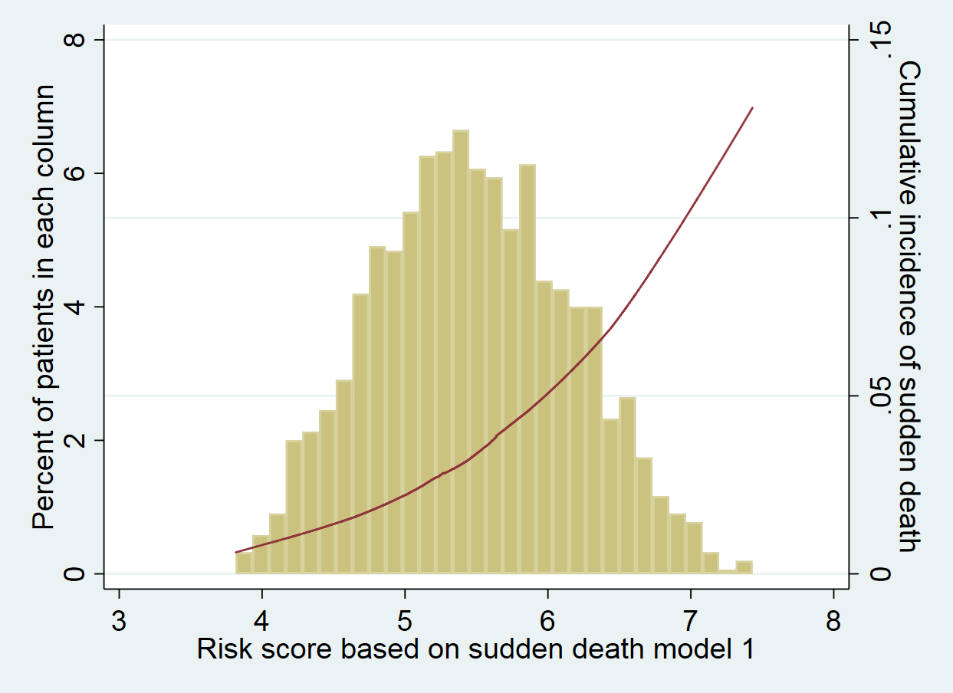

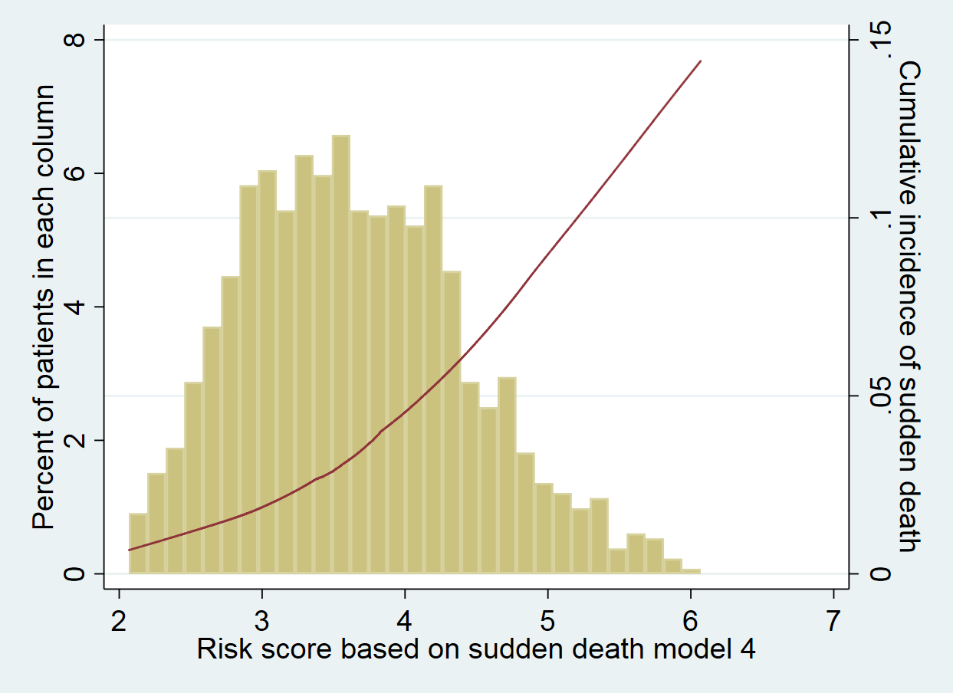


**B**

**A**

Panel A. The risk score and the corresponding cumulative incidence based on Sudden death model 1. Panel B. The risk score and the corresponding cumulative incidence based on Sudden death model 4.

The columns are the histogram of the risk score for sudden death, the left axis shows the percent of patients in each column. The red line is the cumulative incidences of sudden death for the corresponding risk scores.

Online Fig. A9 Distribution of risk score for pump failure death and its relation to cumulative incidence of pump failure death within 4 years in I-Preserve.


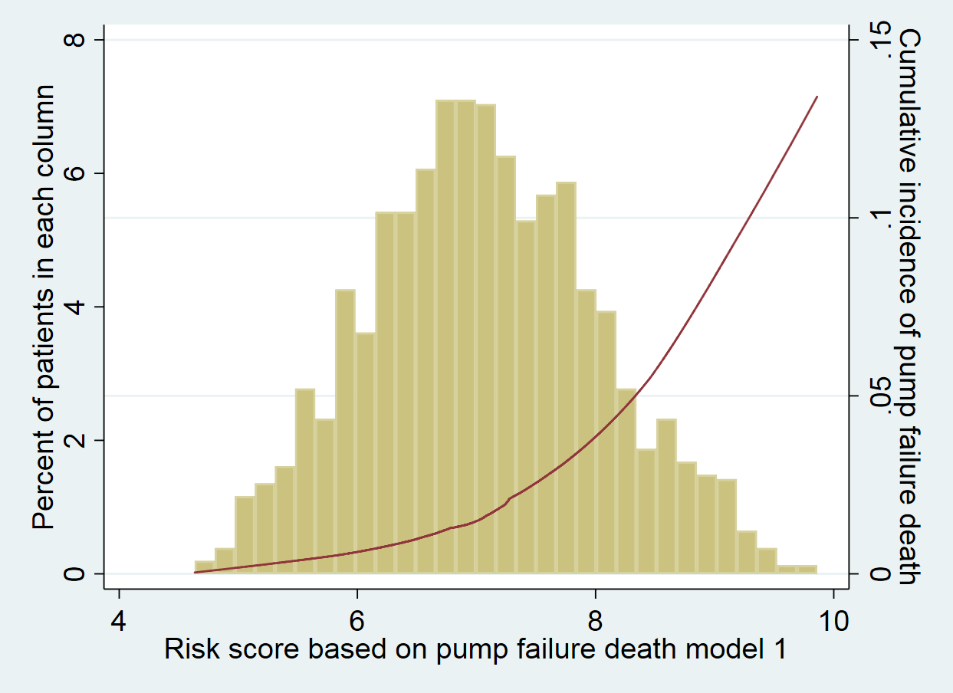

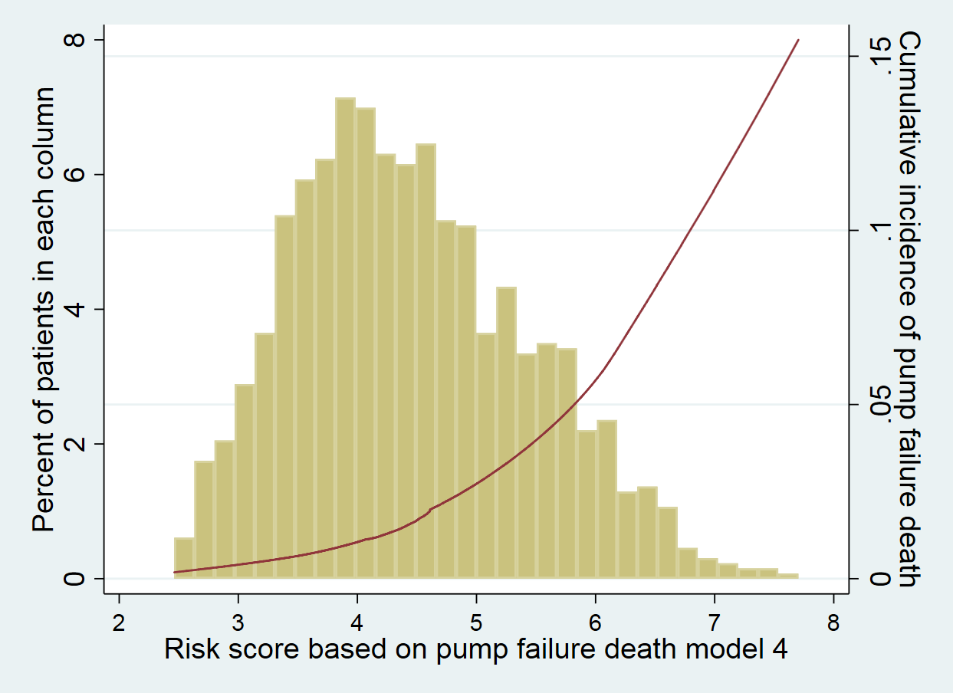


**B**

**A**

Panel A. The risk score and the corresponding cumulative incidence based on Pump failure death model 1. Panel B. The risk score and the corresponding cumulative incidence based on Pump failure death model 4.

The columns are the histogram of the risk score for pump failure death, the left axis shows the percent of patients in each column. The red line is the cumulative incidences of pump failure death for the corresponding risk scores.

**Online Supplement Examples of risk prediction for mode-specific death using the model 4 presented in Table 2 and 3.**

Consider a 72-year-old man, who has HF with preserved ejection fraction with a LVEF of 55%, his baseline diastolic blood pressure is 95 mmHg, he has diabetes with no other comorbidities, and he has no abnormalities on ECG. The serum creatinine level is 1.6 mg/dL and the plasma NT-proBNP is 1400 pg/mL.

Based on the sudden death model 4 in Table 2, the risk score of sudden death is 0.034*72 + 0.506+(60-55)*0.036+0.568+ ln(1400/100)*0.048 = 3.83. Supplemental Figure H indicates that this patient has a 4% probability of sudden death within 4 years.

Based on the sudden death model 4 in Table 3, the risk score of pump failure death is 72*0.044+ 0.758 + 1.6*10*0.060+ 1400/100*0.059 = 5.71. From Supplemental Figure I, we can see this patient has 4.5% chance of pump failure death within 4 years.
